# Dynamic Protein Structure Paradox: An Integrative Framework for Endpoint-Conditioned Evidentiary Sufficiency in Structure-to-Function Claims

**DOI:** 10.3390/cells15171560

**Published:** 2026-08-28

**Authors:** Sarfaraz K. Niazi

**Affiliations:** College of Pharmacy and Pharmaceutical Sciences, Washington State University, Spokane, WA 99202, USA; s.niazi@wsu.edu

**Keywords:** structure–function relationship, conformational ensemble, model credibility, evidence assessment, scoring rubric, structural genomics, intrinsic disorder, protein structure prediction, AlphaFold, functional inference

## Abstract

**Highlights:**

**What are the main findings?**

- Structural accuracy for a represented molecular state and evidentiary sufficiency for a specified functional endpoint are separate properties, and the Dynamic Protein Structure Paradox (DPSP) framework separates them using four coupled dimensions: relevant state, context, ensemble or kinetics, and chemistry.
- The framework is operationalized as a published scoring rubric with explicit adequate, uncertain, and missing criteria; worked examples; a materiality test; three mutually exclusive modes of use; and prespecified conditions that would refute it.

**What is the implication of the main finding?**

- Authors, reviewers, and decision-makers can state, in one sentence per claim, what a structure supports, which decisive variable remains unmeasured, and which corroboration would close the gap.
- Because the rubric and its thresholds are provisional operational decisions, the framework’s value relies on the reliability and incremental validity studies as outlined, rather than on the argument’s plausibility.

**Abstract:**

Accurate coordinates for a represented protein state do not, by themselves, establish activity or any other condition-specific function. This article defines the Dynamic Protein Structure Paradox (DPSP) as the apparent conflict between structural accuracy and functional underdetermination and develops it as an integrative evidentiary assessment framework rather than a new theory or paradigm. The underlying problem has been longstanding, since structural genomics, function annotation, allostery, and disorder research each established that fold does not determine function and that function does not determine fold. DPSP consolidates those results into one endpoint-conditioned rule. Once a measurable endpoint is defined, it assesses four coupled dimensions: relevant-state completeness, context completeness, ensemble or kinetic dependence, and chemical dependence. A rubric rates each dimension as adequate, uncertain, or missing, and a materiality test determines which gaps influence the stated decision. The outcome is one of three mutually exclusive modes of utilization: geometry-led, conditional, or function-measured. The deliverable is a concise evidence statement delineating what the structure supports, which decisive variable remains unmeasured, and what corroboration is necessary. DPSP complements, rather than replaces, existing structural, ensemble, and computational approaches. The framework remains unvalidated, its thresholds are provisional, and the studies necessary to confirm or refute it are specified.

## 1. Introduction

### 1.1. Structure as a Powerful but Conditional Representation

The structure of myoglobin and the subsequent development of the Protein Data Bank have established three-dimensional structure as a fundamental language of molecular biology [1,2]. Structural analysis discloses folds, domain organization, catalytic residues, ligand pockets, interfaces, and steric constraints. Artificial intelligence systems have significantly broadened access to this information, with AlphaFold, RoseTTAFold, ESMFold, and AlphaFold 3 now producing precise coordinate models for a vast array of proteins and biomolecular complexes [3,4,5,6].

The interpretive problem is narrower than the technical achievement. A coordinate model is sometimes used to support a claim pitched at a different level of description: that a protein has a particular activity, affinity, rate, selectivity, stability, response to an effector, or cellular phenotype. Structural information is often highly informative for such questions, but the inference is conditional rather than automatic. Function prediction has always required sequence context, evolutionary evidence, biochemical knowledge, and experimental confirmation beyond geometry [7]. The evidentiary gap treated here opens whenever the accuracy of a represented structure is taken as equivalent to the accuracy of a particular functional claim.

### 1.2. The Problem Is Long-Standing, and This Article Is an Integration Rather than a New Paradigm

The discrepancy between structural information and functional annotation is not a novel discovery of the prediction era. Comparative structural analysis conducted decades ago demonstrated that a limited number of superfolds account for a significant proportion of resolved structures, thereby indicating that fold recurrence provides limited functional insight [8]. A systematic examination of enzyme superfamilies revealed substantial functional diversity among structurally related proteins, with functional divergence occurring often below approximately thirty percent sequence identity [9]. Conversely, moonlighting proteins exemplify a situation where a single polypeptide performs unrelated functions without any alteration in fold [10]. It is important to note that neither the hypothesis that a common fold implies a unique function nor the hypothesis that a particular function necessitates a specific fold is universally applicable.

The international structural genomics programs emphasized this point on a broad scale. From 2000 to 2015, the Protein Structure Initiative and its European and Japanese counterparts established pipelines for target selection, protein production, crystallization, and structure determination, subsequently delivering over thirteen thousand structures predominantly from families lacking prior structural coverage [11,12]. These programs also yielded conceptual advances, including a clearer understanding of fold reuse, superfold distributions, and evidence of convergent evolution toward similar active-site chemistries from unrelated scaffolds. A significant practical conclusion was that assigning function to a newly determined structure without additional experimental verification posed a major challenge. Furthermore, reliable annotation required integrating evidence from sequence analysis, conserved functional-site templates, and biochemical assays [7,13]. Consequently, structural genomics anticipated, within its own framework, the core issue that this article seeks to formalize.

This history informs the way the present contribution should be interpreted. DPSP serves as an integrative framework that consolidates established findings from structural genomics, function annotation, allostery, conformational-ensemble analysis, intrinsic disorder, and structure-guided drug discovery into a single operational principle suitable for the current context, where precise coordinates are inexpensive, plentiful, and readily adaptable to functional discourse. It is not asserted that a novel physical principle has been discovered. The comparison made below with the Anfinsen and Levinthal paradoxes is purely expository. It signifies a third stage of inferential reasoning and delineates three distinct mappings for the reader’s understanding. It does not claim equivalence in mechanistic scope, mathematical formulation, or empirical validation, and the core argument of this article remains unaffected by this distinction.

### 1.3. Formal Statement of the Dynamic Protein Structure Paradox

The DPSP can be stated formally. An experimentally determined or computationally predicted structure may be precise for a particular molecular state; however, the function of that molecular species under specified conditions remains non-unique or only partially inferable from the coordinates. Both assertions coexist because they pertain to distinct entities. Structural accuracy concerns conformity with a represented state, whereas functional sufficiency concerns whether that state, its population, its transitions, relevant chemistry, and biological context influence the specified endpoint.

The term “paradox” signifies an apparent contradiction that resolves itself once an underlying distinction is identified. In Anfinsen’s context, this distinction lies between the thermodynamic preference encoded by the sequence and the trajectory followed to attain the native conformation [14]. In Levinthal’s context, it concerns the difference between an exhaustive random search and a biased movement across a structured energy landscape [15]. In the present scenario, it contrasts the accuracy of a structural depiction with its sufficiency for a functional assertion. The three mappings extend from sequence to structural preference, from conformational search to folding duration, and from the represented structure to functional evidence. These mappings are sequential rather than competing, and they are not mechanistically equivalent.

### 1.4. Scope, Contribution, and Relation to Existing Frameworks

Several established frameworks already require a defined question, an assessment of decision consequences, and evidence scaled to model risk. The FDA framework for artificial intelligence supporting regulatory decisions and ASME V for computational models in medical devices both operate this way [16,17]. The Critical Assessment of Function Annotation evaluates function prediction against specified functional targets using prospective benchmarks [18,19], and SKEMPI 2.0 provides an endpoint-specific benchmark for mutation-induced changes in protein–protein binding [20]. Because a reasonable reader may ask whether an additional framework is warranted, Table 1 states directly what each of these evaluates, what each leaves unaddressed for the present problem, and what DPSP adds.

The asserted contribution is intentionally narrow. DPSP distinguishes between the accuracy of a represented molecular state and the evidentiary adequacy for a specific functional endpoint, identifies four protein-specific sources of missing evidence, provides a rubric that evaluates each source based on predefined criteria, and delineates the conditions under which these evaluations would be considered to contain no information. Energy-landscape theory and conformational-selection models elucidate the existence of multiple states but do not specify the evidence required for a particular functional assertion. Integrative and generative ensemble methods offer more detailed structural descriptions, whereas DPSP operates on the available description and assesses whether it is sufficient for a designated endpoint. Model-confidence metrics measure the internal reliability of a coordinate prediction, which differs fundamentally from functional sufficiency. Furthermore, this article differs from the earlier risk-tiered framework discussed in Section 9, which assesses validation burden across artificial intelligence applications rather than analyzing the informational gap between a representation and a claim.

This article presents a perspective grounded in established research rather than a systematic review or meta-analysis. It does not quantify how often published structure-to-function assertions lack decisive corroboration, nor does it include a retrospective outcome audit. The illustrative scoring detailed in Section 5 exemplifies rubric mechanics conducted by a single assessor without blinding, and should not be construed as evidence of reliability, predictive validity, or prevalence. Throughout the article, specific protein systems are referenced to demonstrate how the assessment proceeds and to identify potential gaps; these references are not accusations that investigators failed to provide required evidence.

## 2. Physical and Informational Basis of the Paradox

### 2.1. Sequence, Folding Landscapes, and Native Ensembles

**Figure 1:**
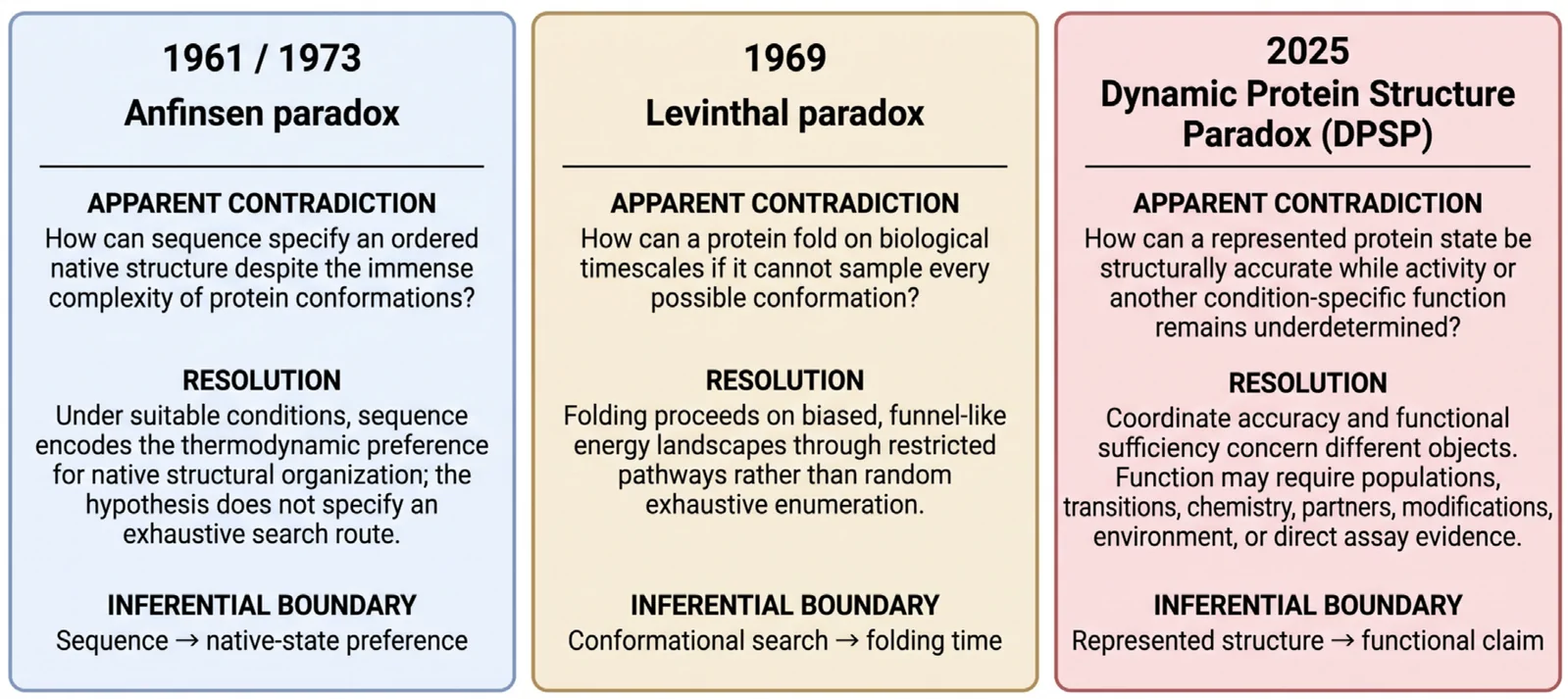
Three distinct inferential mappings in protein science. The dates indicate Anfinsen’s foundational experiments and formal statement, Levinthal’s published formulation, and the 2025 DPSP formulation of the structure-to-function problem [29,30]. The panels are aligned for exposition only. The three formulations concern different mappings, are not mechanistically equivalent, and are not presented as three members of one class; the DPSP panel states an evidentiary boundary rather than a physical principle. Abbreviation: DPSP, Dynamic Protein Structure Paradox.

Anfinsen demonstrated that, under appropriate conditions, the amino acid sequence contains the information necessary to attain the native structure [14]. Levinthal noted that folding cannot be achieved solely through exhaustive enumeration of conformations [15]. Energy-landscape theory addressed the apparent kinetic contradiction by substituting random searches with biased movements across a rugged landscape [24,25,26,27]. The DPSP process begins after the sequence-to-structure problem. Even when a state has been accurately identified, measured, or predicted, reverse inference from that state to a condition-specific function may still be non-unique. Figure 1 illustrates the three sequential mappings.

The sequence remains the primary determinant of accessible structure, while the populated ensemble depends on temperature, solvent, protonation, redox state, ligands, cofactors, oligomerization, post-translational modifications, and cellular machinery. Chaperones alter folding kinetics and suppress off-pathway states without changing the underlying thermodynamics. The conclusion required for DPSP is modest: sequence defines possibilities and energetic preferences, while a biological experiment observes the subset populated under specified conditions. Protein dynamics across timescales supply the established physical basis for this distinction [28].

### 2.2. Why an Accurate State Can Be Functionally Insufficient

The function depends on physical determinants that vary across cases. Some questions depend mainly on geometry, such as whether a folded domain encloses a cavity of a specific shape. Others are contingent upon populations and transition processes, exemplified by conformational selection and allostery; chemical steps and electrostatic preorganization, as observed in catalysis; association and dissociation kinetics, exemplified by residence time; or solution behavior, including aggregation and colloidal stability [31]. These determinants are distinct and should not be consolidated into a single hierarchy.

A structure is therefore adequate solely with respect to a designated endpoint. Coordinates pertaining to a ligand-bound state support hypotheses regarding binding poses but do not establish equilibrium affinity, association rate, dissociation rate, or cellular efficacy. A ground-state enzyme structure can define catalytic residues and metal coordination without determining the rate-limiting step or the turnover number. A high-resolution antibody–antigen complex can identify an epitope without resolving avidity, polyspecificity, developability, or in vivo potency. The critical question shifts from whether the structure is accurate to whether this representation suffices for the specific claim being made. This distinction is elaborated here and then applied, rather than reiterated, in subsequent sections, which apply it to specific cases without restating the underlying argument.

### 2.3. Functional Inference as an Underdetermined Inverse Problem

The forward biological mapping progresses from sequence and context through a conformational ensemble, kinetics, and chemistry to an observed function. Experimental structure determination samples or averages states under measurement conditions, whereas computational prediction infers coordinates from learned sequence-structure relationships and supplied inputs. Both approaches produce a structural representation, using different methodologies and facing distinct limitations. Inferring function in the reverse direction from a coordinate set constitutes an inverse problem. This is manageable when the endpoint is predominantly influenced by features represented by the structure and when alternative explanations are limited. Conversely, it becomes underdetermined when distinct unobserved ensembles or contexts can generate the same structural representation yet yield different functional outcomes.

**Figure 2:**
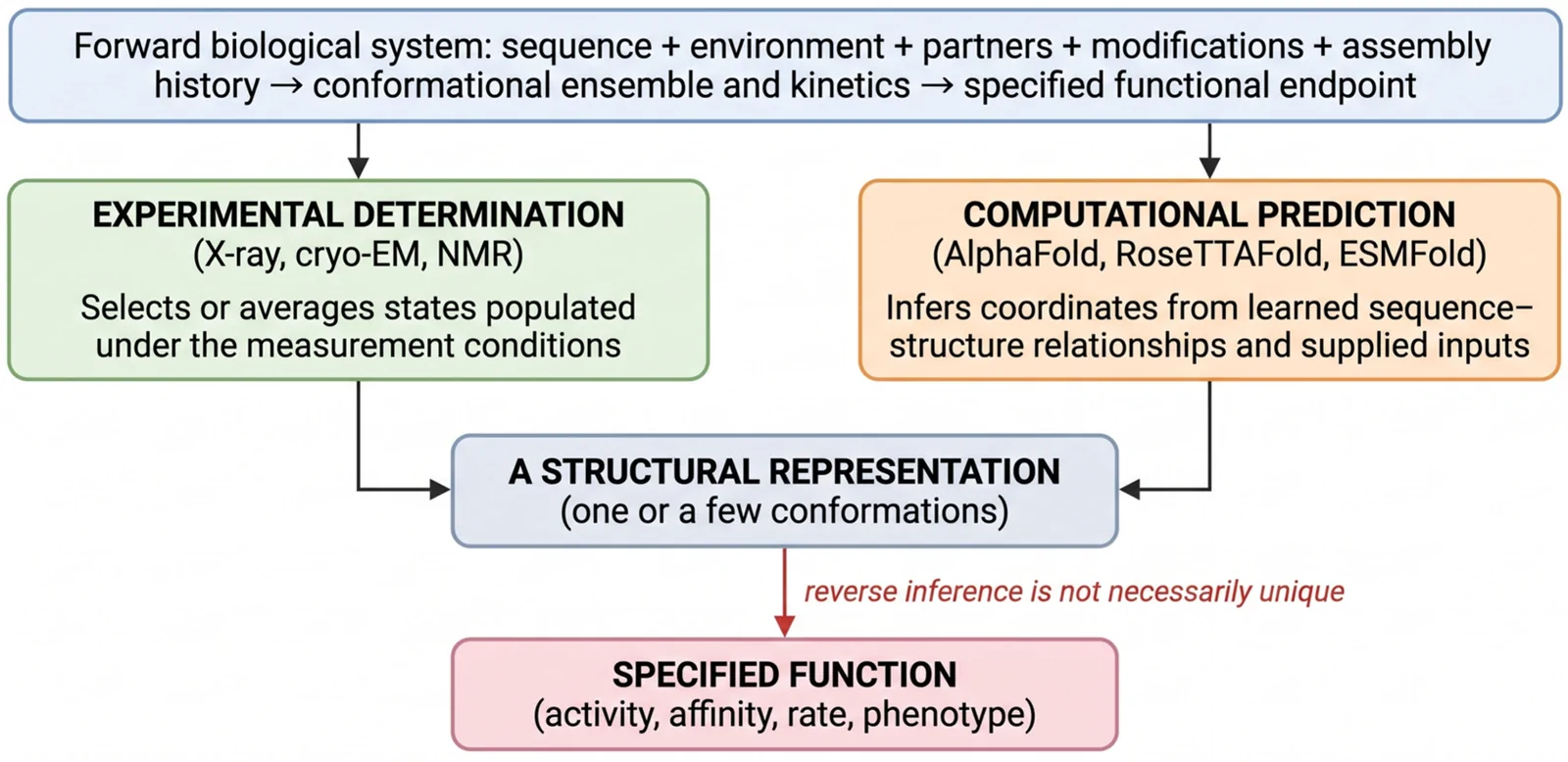
Forward and reverse mappings essential to the Dynamic Protein Structure Paradox (DPSP). A biological system, comprising sequence, environment, partners, modifications, and assembly history, produces an ensemble and kinetics that support a designated functional endpoint. Both experimental determination and computational prediction serve as methods for deriving a structural representation, which may encompass one or a few conformations. Reverse inference from this representation to the specified function is not necessarily unique, as critical variables such as state, population, kinetics, chemical, or contextual factors may be absent. This is a conceptual schematic; no empirical data are provided.

This framing also separates thermodynamic heterogeneity from epistemic uncertainty. A protein may genuinely occupy many states, which is a property of the system, while knowledge of those states and their populations may be strong or weak, which is a property of the evidence. DPSP addresses both. Broad ensembles can make single-state inference incomplete, and limited knowledge can complicate interpretation even of a narrow ensemble. The framework therefore does not equate flexibility with ignorance and does not assume that every flexible region is functionally significant. Figure 2 summarizes the non-unique reverse inference.

### 2.4. Structural Added Value Relative to Sequence and Evolutionary Information

A structure-to-function assessment remains incomplete unless it specifies the structure’s unique contributions that are not obtainable from less costly sources. Structural added value is herein characterized as information pertinent to the designated endpoint, which exists within the coordinate model and is absent from the sequence, its alignment, and standard annotation. This definition is more rigorous than the informal usage, wherein any three-dimensional representation is regarded as added value.

Two categories should be distinguished. The first involves recapitulating structural information, which reiterates data provided by sequence and evolutionary analyses. Assignments of protein folds, domain boundaries, and the identification of conserved catalytic residues predominantly fall within this category. The emphasis here is quantitative rather than rhetorical, as evolutionary couplings derived from extensive alignments have constrained tertiary contacts and yielded approximate folds well before end-to-end learned predictors emerged. Moreover, deep metagenomic alignments extended these findings to thousands of protein families [32,33,34]. Additionally, alignment-based statistical models can predict mutational effects without reliance on coordinate data [35]. Because much of the accuracy of current predictive models depends on this evolutionary signal, a predicted structure of a well-aligned globular domain often merely restates information inherently contained in the alignment.

The second category concerns non-redundant structural information that cannot be reconstructed through sequence analysis at comparable resolution. This includes attributes such as the shape, volume, and electrostatic properties of a ligand-binding pocket; the geometry and buried surface area of a protein–protein interface; spatial adjacency among residues distant in the sequence; potential allosteric pathways traversing densely packed cores; and local structural effects from substitutions. Explicitly articulating these relationships constitutes a significant contribution rather than merely facilitating visualization, as it transforms a list of conserved positions into a three-dimensional geometry that can be utilized for docking, designed mutations, and experimental validation. The framework recognizes this class as substantive evidence and refrains from undervaluing it.

The consequence for assessment is direct. The question is not whether a model is static but whether the represented state supplies the specific information the endpoint requires and whether that information is unavailable from cheaper sources. A model can be complete with respect to the relevant state and still add little, if the endpoint is answerable from the alignment. A model can add a great deal and still fail on ensemble or chemical dependence. Where a claim is settled by sequence-level evidence alone, the correct assessment is that structure is not the decisive evidence in that instance, not that structure is deficient.

## 3. Evidence for Ensemble- and Context-Dependent Function

### 3.1. Solution and Single-Molecule Measurements

Nuclear magnetic resonance relaxation and exchange experiments provide insights into molecular motions and low-population states across various timescales [36]. Hydrogen-deuterium exchange mass spectrometry offers information regarding regional protection, solvent accessibility, and conformational opening, although exchange time is not universally indicative of the underlying motional timescale [37]. A recent multiplexed study analyzed 5778 domains ranging from 28 to 64 residues and used site-resolved measurements on 13 domains, showing that sequences sharing a common fold and similar global stability may nonetheless differ in conformational fluctuations [38]. Single-molecule fluorescence techniques resolve trajectories and subpopulations that are otherwise obscured by bulk averaging [39]. These methodologies provide complementary insights into different aspects of an ensemble and should not be considered interchangeable.

No single method, therefore, possesses exclusive access to the functional ensemble. Nuclear magnetic resonance is limited by molecular size and exchange regime; hydrogen-deuterium exchange is constrained by spatial resolution and model dependence; and single-molecule techniques are restricted by labeling prerequisites and observable selection. Their value resides in their complementarity. When multiple observables concur on the same state distribution or transition, the inference becomes more robust than when flexibility is deduced from a solitary indirect proxy.

### 3.2. Structural and Computational Methods Also Resolve Heterogeneity

Structural methodologies inherently address conformational heterogeneity; thus, the distinction between structure and ensemble pertains to informational content rather than a dichotomy between structural and non-structural sciences. Room-temperature crystallography and ensemble refinement elucidate alternative sidechain and backbone conformations within crystalline structures [40]. Cryo-electron microscopy classification and variability analysis differentiate discrete states from continuous motions in particle images [41]. Long-timescale unbiased molecular dynamics simulations, complemented by enhanced sampling techniques such as metadynamics, help formulate mechanistic hypotheses about transitions and free-energy landscapes [42,43]. Integrative ensemble determination amalgamates simulation data with experimental observables, avoiding reliance on either alone as conclusive evidence [22]. Consequently, DPSP broadens the scope of dynamic structural biology and applies it to a single question: whether a specific coordinate model provides sufficient evidence for a specified functional assertion.

Computational ensembles demand the same discipline as experimental structures. Sampling may be incomplete, force fields or learned distributions may be misspecified, and computed populations might not accurately represent the thermodynamic conditions of interest. Consequently, validation against independent observables is more critical than merely increasing the number of generated conformations. A large ensemble that excludes a rare functional state can be less valuable than a smaller, experimentally supported set that includes it.

### 3.3. Disorder as an Alternative Mode of Functional Organization

Intrinsically disordered regions are often regarded as a challenge in structure prediction. However, this perspective is incomplete and not the one adopted herein. Disordered regions encompass significant sequence-encoded functional information. Short linear motifs facilitate processes such as targeting, degradation, docking, and modification through compact, partially conserved elements [44]. Molecular recognition features undergo coupled folding and binding, accounting for a substantial portion of regulatory interactions [45,46]. Segments rich in modifications encode reversible regulatory logic, with sequence grammars, including the valence and patterning of aromatic residues, governing phase behavior and condensate formation [47,48]. These functions are inherent properties of the sequence, supporting the central thesis of this article: function is organized in a way that no single stable three-dimensional structure can fully represent.

Two consequences emerge concerning assessment. Firstly, disorder is recognized independently of structure prediction. Community blind assessment evaluates disorder predictors against curated experimental annotations, and the most effective methods perform substantially above physicochemical baselines [49]. Consequently, low model confidence should not be treated as primary evidence of disorder or used as a proxy; it may also result from shallow alignments, ambiguous domain orientations, and other factors [50]. Secondly, the inability to assign a single stable structure pertains to the representation, whereas intrinsic disorder pertains to the molecule itself. These must therefore be documented separately. Within the DPSP framework, a disordered region is not considered a defect. Instead, it is a region where functional information is contained within recognition elements and condition-dependent state distributions, which situates the related assertion within the context and ensemble dimensions rather than under pertinent-state geometry.

### 3.4. Allostery, Fold Switching, and Cellular Context

Classical and contemporary conceptions of allostery depend on the redistribution among pre-existing states, ligand-induced conformational modifications, or dynamic coupling mechanisms, rather than on a singular conformation [51,52]. G-protein-coupled receptors exemplify how ligands stabilize distinct active and inactive ensembles, thereby modifying signaling outcomes [53]. Fold-switching proteins represent a more pronounced example, as a single sequence can support multiple structured states, each with different functional roles [54].

The intracellular environment further influences the interpretation of isolated structures. Macromolecular crowding impacts association and conformational equilibria [55,56], and chaperone systems regulate folding pathways and proteostasis [57]. In-cell nuclear magnetic resonance has demonstrated that structure, interactions, and maturation can be observed within living human cells [58,59]. Glycans, membranes, metals, and other partners stabilize or alter states absent from an apo model, and specialized glycoprotein modeling exists because protein backbone coordinates do not solely determine the glycan ensemble [60].

Non-protein partners are therefore not merely a marginal special case; rather, they are variables that influence the admissibility of a structural claim concerning an endpoint. Understanding their roles helps pinpoint their positions within the assessment. Nucleic acids and chromatin operate through both dimensions of completeness, because a DNA- or RNA-binding protein frequently undergoes a disorder-to-order transition upon binding and interprets sequence-dependent nucleic acid conformation. Consequently, the bound nucleic acid qualifies as relevant-state completeness, while its sequence, modifications, and chromatin context are categorized as contextual variables [61]. Glycans and glycoforms serve as relevant-state variables when a specific glycan is essential for the functional complex; conversely, they function as contextual variables when glycoform distribution influences clearance or recognition. A deglycosylated backbone is deemed incomplete in either scenario [60]. Membrane composition, curvature, and lateral pressure primarily fall under context, since the same membrane protein adopts different conformational states in different lipid environments. However, a tightly bound structural lipid is classified as a relevant state [62]. Metals, cofactors, and metabolites are chiefly relevant-state variables because the correct metal ion or cofactor dictates coordination geometry and catalytic organization, whereas their cellular availability constitutes a contextual factor [63]. In each instance, the fundamental inquiry remains: Is the functional molecular species complete? Are the partners or conditions that facilitate the relevant state adequately represented?

### 3.5. Bounded Applicability: When a Single Structure Is Highly Informative

The existence of ensembles does not deprive individual structures of their value. A single structure can demonstrate fold and domain architecture, identify sterically accessible pockets, reveal catalytic residues, support mechanistic hypotheses concerning disease-associated mutations, map interfaces, and guide construct design. It can serve as a sufficient working representation for geometric inquiries when the relevant state, molecular composition, and environment are known. The limitation is not that proteins are uniformly dynamic; rather, it is that a structural model should not be ascribed information it does not contain.

Bounded sufficiency includes a rigid interface captured in the correct complex, a conserved active-site geometry whose chemical role is independently established, and a pocket whose presence is robust across experimentally supported states. Aggregation, misfolding, and transient exposure of hydrophobic surfaces, by contrast, can depend on low-population conformations invisible in the dominant structure [64]. Table 2 separates common functional questions without treating them as commensurate.

## 4. The DPSP Framework

### 4.1. Define the Functional Endpoint and Place It in the Evidence Hierarchy

The first step is to replace the term “function” with a quantifiable endpoint. Binding may refer to a conformation, an equilibrium dissociation constant, association and dissociation rates, competition, avidity, or neutralization. Catalysis may denote substrate recognition, turnover number, catalytic efficiency, regioselectivity, or product release. Stability may encompass equilibrium unfolding, kinetic stability, shelf life, resistance to agitation, or aggregation at a specified concentration. One structural measurement may suffice for one parameter but be inadequate for another.

The endpoint must specify the molecular species and the conditions pertinent to the claim. A statement addressing an apo monomer at neutral pH does not pertain to a ligand-bound oligomer within a membrane or to a glycosylated protein in serum. This requirement primarily safeguards against category errors and prevents attaching fixed labels such as ‘structurally predictable’ or ‘dynamics dependent’ to a protein when different activities of the same protein require distinct evidence. Additionally, the endpoint must clearly articulate the intended decision and the consequences of potential errors, as evidence sufficient for hypothesis generation may not be adequate for candidate selection, mechanism verification, or regulatory claims. The required level of corroboration correlates with the risk associated with the decision, not the protein itself.

Endpoints are not interchangeable, and it is advantageous to categorize them according to the amount of information beyond mere geometry that each necessitates. Level 1 pertains to the represented structural state, encompassing fold, composition, and conformation. Level 2 relates to molecular interactions, including binding pose, contacts, and interface. Level 3 comprises thermodynamic and kinetic parameters, such as equilibrium dissociation constants, association and dissociation rates, mutation-induced alterations in binding energy, and residence time. Level 4 involves biochemical consequences, including turnover, inhibition, selectivity, and stability outcomes. Level 5 pertains to cellular and organismal phenotypes. Structural evidence is most compelling at Levels 1 and 2, somewhat present at Level 3, and generally inadequate on its own at Levels 4 and 5.

The levels are ordered by their evidential proximity to geometry, not by importance, and are not arranged linearly. A significant Level 3 change may not alter a Level 5 phenotype due to receptor reserve, pathway compensation, or buffering mechanisms, whereas a minor Level 2 change, such as a single resistance substitution, can dominate the outcome at Level 5. Consequently, the hierarchy serves as a tool for identifying an endpoint and predicting which dimensions will be pertinent, rather than as a chain of causality. Additionally, it clarifies the division of responsibilities among the tables presented in this article. Table 2 is organized by question type and delineates what a structure can support for that question. Table 3 is organized by the source of potential missing evidence and defines the assessment dimensions. Table 4 outlines the rating criteria for these dimensions, while Table 5 provides practical examples for each rating. These four tables complement each other rather than serving as alternative versions of a single list.

### 4.2. Relevant-State Completeness

Relevant-state completeness examines whether the model encompasses the molecular state responsible for executing the endpoint. The evaluation includes conformational state, oligomeric state, ligand or substrate presence, cofactor or metal involvement, mutation, protonation state, and post-translational modifications. An accurate apo structure alone does not constitute comprehensive evidence for a function performed by a holo complex. A monomer model is insufficient for an obligate oligomer. A structure missing a regulatory domain or a flexible linker cannot support claims that rely on those components.

Completeness does not necessarily equate to overall model quality. A model may exhibit exemplary global geometry while failing to include the specific state that executes the function. Furthermore, a locally uncertain segment may be irrelevant to an alternative endpoint. Consequently, the assessment primarily concentrates on the functional region and its contacts, rather than relying on an aggregate confidence score across the entire chain.

### 4.3. Context Completeness

Context completeness inquires whether the conditions that determine or stabilize the functional state are sufficiently represented. Relevant variables encompass pH, ionic strength, redox potential, membrane composition, molecular crowding, ligand concentration, competing partners, glycosylation, phosphorylation, proteolytic processing, and cellular compartment. The list is limited to variables with a mechanistic basis for influencing the endpoint, as the framework does not require reconstructing an entire cell for each inquiry.

In the absence of context, the appropriate response is to refine the claim rather than dismiss the structural framework. An apo structure corroborates the apo-state geometry; however, without additional evidence, it cannot determine the population or activity of a ligand-selected state. Consequently, context determines the scope of interpretation rather than diminishing a general confidence score.

### 4.4. Ensemble, Kinetic, and Chemical Dependence

Ensemble dependence matters when the endpoint involves redistribution among states, transient exposure, disorder-to-order transitions, or a low-population conformation. Kinetic dependence is substantial when association, dissociation, gating, exchange, or turnover rates influence the endpoint. Chemical dependence is prominent when processes such as proton transfer, electrostatics, solvent organization, or transition-state stabilization govern the measured activity. Enzyme catalysis exemplifies that these mechanisms can operate simultaneously, as a preorganized active site, conformational gating, and chemical steps may all contribute; therefore, flexibility should not be presumed to be the sole source of rate enhancement [65,66]. Nonetheless, alternative conformations can be essential to a catalytic cycle [67].

The question is not whether motion exists, since thermal motion is universal. The question is whether unaccounted populations or transitions would change the conclusion. A variable is material when plausible values would change the decision within the prespecified error tolerance. Evidence may come from exchange measurements, hydrogen-deuterium exchange, single-molecule distributions, cryo-electron microscopy classes, room-temperature crystallography, time-resolved methods, mutational coupling, or validated simulation. Not every measurement is needed in every case.

### 4.5. The Four Dimensions Are Coupled, Not Independent

**Figure 3:**
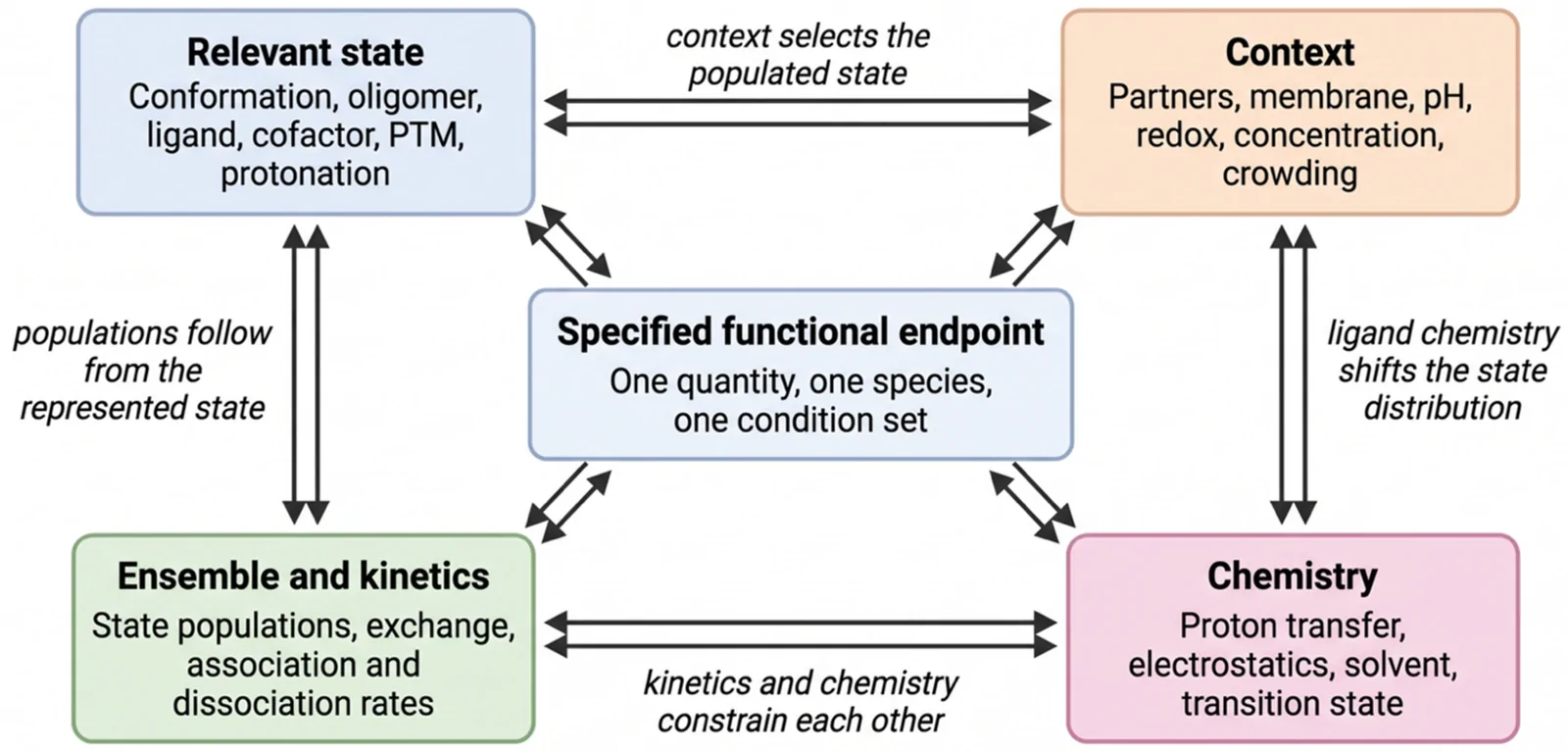
Interconnections among the four DPSP dimensions. Context determines which states are populated; populations govern the apparent kinetics; ligand chemistry influences the distribution of states; and chemistry and exchange rates mutually constrain each other. Diagonal couplings between relevant states and chemistry, as well as between context and the ensemble, are explicitly presented for clarity. Since these dimensions are interconnected, they function as separate accounting categories that inform different corroborating measurements, rather than being combined into a single composite score. This diagram serves as a conceptual framework; no empirical data are included.

The four dimensions constitute distinct accounting categories rather than autonomous physical variables, and treating them as orthogonal would misrepresent protein behavior. Context influences the distribution of state populations, and alterations in these populations subsequently affect the apparent kinetics. Ligand chemistry shifts conformational populations, thereby modifying the measured rates. Furthermore, the context determines the occupancy of post-translational modifications, oligomerization, membrane association, and crowding, each of which reshapes the ensemble. Chemistry and kinetics mutually constrain each other whenever a chemical step is partially rate-limiting. Figure 3 illustrates the principal interactions, including the diagonal relationships between relevant states and chemistry, as well as between context and ensemble.

Coupling possesses two practical implications that are inherently integrated into the procedure. First, the dimensions are never aggregated into a composite score, as coupling would count one physical cause multiple times, producing a number without a well-defined meaning. The framework provides a mode assignment and an evidence statement, rather than a scalar value. Secondly, when two coupled dimensions are both associated with uncertainty, the corroboration should focus on the upstream variable, which is typically the state or context, because resolving this parameter often simultaneously resolves the downstream dimension. For instance, identifying the glycoform present often addresses both a relevant-state question and an ensemble question; in contrast, measuring an exchange rate on the incorrect glycoform resolves neither.

### 4.6. Classify the Evidentiary Use and Specify the Missing Evidence

Geometry-led utilization applies when the structure serves as the primary indicator: the endpoint is geometric, the pertinent state and molecular composition are depicted, the context supports the assertion, and no unrepresented population, rate, or chemical step is expected to alter the result.

Conditional use applies when the structure remains central, but a presently attainable and validated piece of state, context, population, kinetic, or chemical evidence is required to close a clearly identified gap. An unknown variable is not supporting evidence.

Function-measured use applies when the claim cannot presently be supported by attainable structural, ensemble, kinetic, or chemical-state evidence and therefore requires direct measurement of rate, activity, stability outcome, cellular phenotype, or organismal result. Structural evidence still informs hypotheses, perturbations, and interpretation, but it does not substitute for the decisive assay.

**Figure 4:**
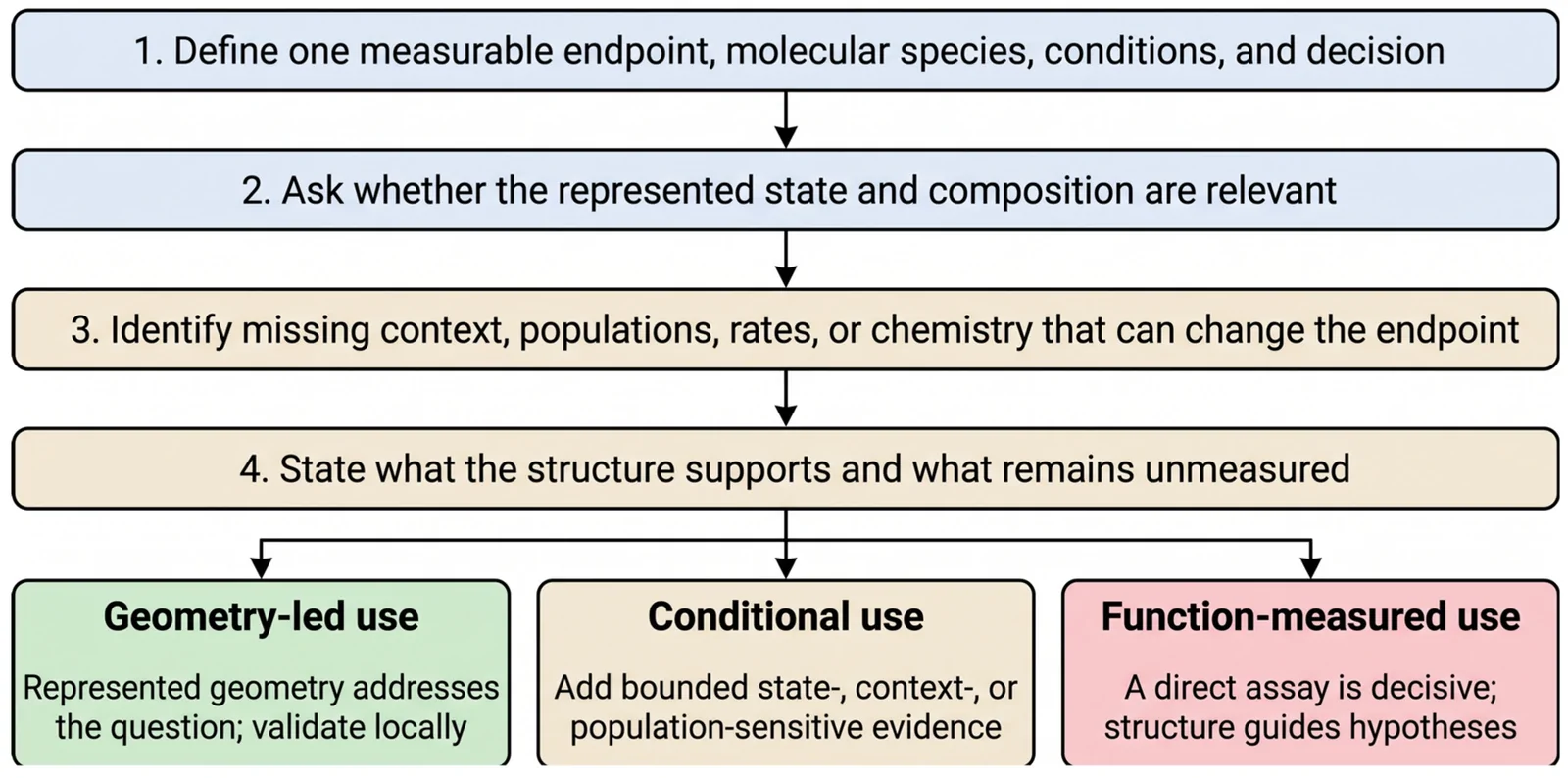
Operational workflow for the implementation of DPSP within a structural model. The process commences with a clearly defined endpoint, followed by an evaluation of the completeness of state and context. Subsequently, it assesses dependence on ensembles, kinetics, and chemistry. The model is then utilized in geometry-led, conditional, or function-measured modes, accompanied by validation procedures suitable to the endpoint. It should be noted that the assignment remains specific to the endpoint and may change if the question varies. This diagram functions as a conceptual schematic; no data are provided. Abbreviation: DPSP, Dynamic Protein Structure Paradox.

The categories are applied in a predetermined sequence. Initially, it must be established whether any unresolved variable is material, which implies that plausible values could alter the stated decision within its predefined error tolerance. If no such variable is material, and the represented state satisfies the bounded geometric inquiry, then geometry-led utilization should be assigned. When a material discrepancy can currently be addressed through attainable, verified evidence, designate conditional use. Conversely, if the material discrepancy cannot presently be remedied by these means and the assertion necessitates direct functional observation, the appropriate designation is function-measured use. The specific identity of the affected dimension does not influence the mode; rather, it determines the corroboration required and the reporting process. These decision rules are mutually exclusive, not part of a calibrated scoring system. Table 3 provides a summary of the endpoint prerequisites and the four dimensions, while Figure 4 illustrates the workflow.

### 4.7. Worked Example: A Fold-Switching Protein

KaiB, a circadian clock protein that interconverts between two fully folded conformations with distinct functions, exemplifies the process. It serves as a valuable test case for single-structure prediction because it supports more than one native fold from a single sequence [54,68].

- Step 1 delineates the endpoint. The inquiry is not concerned with KaiB’s structural configuration, but rather with whether a candidate model accurately depicts and stabilizes the infrequent fold-switched state responsible for binding KaiC under conditions relevant to the circadian clock. The ground-state conformation is the predominant and more stable form; however, it is not the conformation involved in binding KaiC. Instead, binding necessitates the transient, thioredoxin-like fold-switched state.
- Step 2 evaluates the completeness of the relevant state. A model that represents only the ground state is insufficient for an endpoint that relies on the fold-switched state, regardless of the model’s confidence in the conformation it depicts [68,69].
- Step 3 evaluates the completeness of the context. The configuration of the populated fold depends on binding partners and their concentrations; therefore, an isolated monomer model cannot determine which state predominates in the relevant complex.
- Step 4 assesses ensemble, kinetic, and chemical dependence. The endpoint depends on redistribution toward the rare thioredoxin-like state and on interconversion kinetics that affect circadian timing, so a single coordinate set is inadequate [68,69].
- Step 5 designates the mode. The structure is assigned for conditional use: it generates hypotheses for each fold but must be integrated with state-resolved evidence, such as exchange measurements, native mass spectrometry, or partner-bound structures, before substantiating the functional claim.

The reasoning also elucidates why a percentage of low-confidence residues alone cannot assign a protein to a specific category. A model may exhibit uniform confidence regarding the ground-state fold, yet provide no indication concerning an endpoint contingent upon the fold-switched state. Confidence in a representation does not necessarily imply sufficiency for a functional conclusion. The same methodological approach can be applied, without modification, to other context-dependent systems. For instance, in the case of a G-protein-coupled receptor, the activation status, whether signaling is active or inactive, depends on the predominance of the ligand-stabilized ensemble; thus, a single active-state model alone is inadequate to establish an inactive-state functional claim [53]. Similarly, for a therapeutic glycoprotein, an endpoint such as clearance or effector function depends on the glycoform, which a deglycosylated backbone model does not capture, shifting the claim to a conditional or function-assessed usage [60]. In each scenario, the output remains the same: an explicit, endpoint-specific statement detailing what the structure supports and what independent measurements are still required, rather than a categorical label or a single model-confidence score. This procedure applies to both coordinate predictors such as AlphaFold 3 and ensemble models that approximate equilibrium distributions, although the evidential contribution varies: a coordinate predictor provides a single representation, whereas a generative ensemble supplies a distribution that must be evaluated in relation to the endpoint and its context [6,23].

## 5. Operationalizing the Framework

### 5.1. A Scoring Rubric for the Four Dimensions

A framework that cannot be applied consistently by independent assessors is of no practical utility, and a taxonomy articulated solely in prose invites the very failure it aims to prevent. Consequently, this section establishes the operational definitions to ensure clarity. Each dimension is evaluated as adequate, uncertain, or missing based on the criteria outlined in Table 4, with illustrative positive, negative, and ambiguous cases provided in Table 5. Ratings are determined solely with reference to the endpoint defined in Section 4.1, not to any other criterion; a dimension may be deemed adequate for one endpoint and missing for another within the same coordinate file.

Three procedural rules complete the rubric.

- The first is the evidence rule. A dimension is rated adequate only when at least one of the following applies: the required molecular species or condition is directly represented in the model or in an experimental structure of the same species; an independent measurement establishes the required variable under conditions matching the endpoint; or a simulation reproduces an independent observable that is sensitive to the variable in question. Argument from analogy to a homolog, from general expectation, or from model confidence does not establish adequacy.
- The second is the materiality rule. A dimension rated uncertain or missing is material when plausible values of the unrepresented variable would change the stated decision at its prespecified error tolerance. Nonmaterial gaps are recorded, with the reason, and do not alter the mode. This rule prevents the framework from degenerating into a demand that every variable be measured, which would make every claim function-measured and the framework useless.
- The third principle pertains to the disagreement rule. When two assessors differ by one level on a given dimension, the more conservative value is adopted through a consensus process, and the disagreement is documented. When the discrepancy extends to two levels, a third assessor conducts an adjudication using the same written criteria, and the outcome, along with its rationale, is recorded. The materiality judgment, being binary, does not conform to this scheme; hence, any disagreement regarding materiality is directly referred to a third assessor who determines against the endpoint definition and the plausible range of the unrepresented variable, recording the justification accordingly. If adjudication fails to resolve the disagreement, the issue is considered material by default, favoring the conservative stance, and the case remains unresolved and is reported as such. Consensus values are used for reporting purposes; however, individual ratings and materiality judgments are preserved for reliability analyses, since consensus values are unsuitable for agreement estimation. Agreement on materiality is evaluated based on the classification that influences the assessment, specifically whether the record contains at least one material gap.

### 5.2. Illustrative Application of the Rubric

Table 6 employs the rubric to evaluate nine endpoint and representation pairs derived from well-characterized systems. Two rows use the same receptor and the same coordinate model but differ in endpoints, illustrating where the boundary between conditional use and function-measured use lies. The purpose of this is to demonstrate that the rubric yields distinct assessments for different endpoints on the same protein, that these assessments follow from explicitly stated criteria rather than subjective impressions, and that the resulting evidence statement specifies a particular subsequent measurement. Clarify one convention before interpreting the table, as it influences the ratings. Each row is scored based on the representation specified therein and the evidence that a user employing that representation would consider, rather than against the entire body of published literature on the system. This approach aligns with the framework’s requirements, since the assessment addresses what a given model supports regarding a specific claim, and varying representations of the same protein may receive different scores. The KaiB row is evaluated using a ground-state fold model, wherein relevant state information is absent, notwithstanding the existence of state-resolved experimental evidence for the fold-switched conformation elsewhere, which is cited in Section 4.7. A user referencing this experimental evidence would assign a different rating to the relevant state dimension and would accordingly reassess the remaining dimensions and the mode. Additionally, three limitations should be considered alongside the table: first, the scoring was conducted by a single assessor, the author, without blinding regarding the identity of the systems; second, no inter-rater reliability can be estimated from this process, nor is any claimed; third, the cases were selected to cover the spectrum of assignments rather than being randomly sampled from the literature, and therefore, the table does not provide information regarding the frequency with which published claims lack corroboration. The entries describe specific representations and endpoints, not the claims of any publication. The questions concerning reliability and incremental validity are addressed solely through the prospective studies outlined in Section 8.

## 6. AI-Based Structural Prediction and the DPSP

### 6.1. What Current Structure Predictors Provide

AlphaFold2, RoseTTAFold, and ESMFold generate coordinate models based on sequence-derived data and learned structural regularities. AlphaFold 3 expands this capability to encompass complexes involving proteins, nucleic acids, ligands, ions, and certain modified residues [3,4,5,6]. Its performance is evaluated through concordance with experimental coordinate data and interaction benchmarks, and extensive databases of predicted structures significantly enhance structural coverage across proteomes [74,75].

A balanced account of this success is significant for the argument. Much of the attainable accuracy for well-aligned families relies on evolutionary information, as correlated substitution patterns constrain tertiary contacts, as previously established through end-to-end learned predictors and extended by metagenomic sequence depth [32,33,34]. Consequently, a predicted model does not inherently constitute new information, nor is the question of whether the model is static of primary interest. As outlined in Section 2.4, the pertinent inquiry is what non-redundant information the predicted structure provides relative to sequence and alignment, and whether that information aligns with the endpoint’s needs. Since these models supply coordinates, they do not, by themselves, confirm biological activity. A predicted pocket constitutes a structural hypothesis; a predicted complex signifies an interaction hypothesis; and a high-confidence fold offers evidence regarding architecture. The functional interpretation remains dependent on the endpoint, the state, the context, and corroborating evidence.

### 6.2. Model Confidence Is Not Functional Sufficiency

Per-residue confidence and predicted-error metrics serve as valuable indicators for internal reliability assessments, domain positioning, and interface uncertainty analysis. They do not serve as calibrated indicators of conformational populations, free energies, kinetic properties, or functional characteristics. Community evaluations have documented both the broad applicability of AlphaFold2 and the necessity for caution when interpreting specific domains within complexes, disordered regions, ligands, and alternative conformational states [21]. Conformational diversity can reduce concordance between a single predicted model and multiple experimental conformations with low confidence [76], and regions of low confidence may indicate intrinsic disorder, weak evolutionary signals, ambiguous domain orientations, or other factors [50]. As outlined in Section 3.3, low confidence levels should not be construed as indicative of disorder, which requires independent validation [49].

Comparison with solution nuclear magnetic resonance reveals limitations. Predicted models can closely correspond to well-ordered small proteins and domains [77], whereas a single model may not adequately represent solution heterogeneity, especially in flexible or state-switching regions [78]. High coordinate confidence should therefore be interpreted as an indication that the model is confident in this representation, rather than implying that this representation is adequate for all functional assertions.

### 6.3. Alternative-State and Ensemble Models

Prediction pipelines can be adapted to generate alternative conformations. Reduced or clustered multiple-sequence alignments, repeated seeds, and related techniques have yielded distinct native-like states for transporters and receptors [79]. These approaches are valuable for hypothesis formulation; however, the diversity they produce may not necessarily represent an equilibrium population and can be heavily influenced by the sampling protocol.

Generative equilibrium-ensemble emulation is a distinct methodological category designed to approximate equilibrium structural ensembles for protein monomers rather than predict a single static structure [23]. Its development directly addresses a portion of the gap identified herein. An ensemble model, however, remains dependent on its training data, the force-field-derived information it utilizes, and its assumptions regarding molecular composition and environment; it does not, by itself, establish cellular function, ligand-dependent chemistry, post-translational processing, or the relevance of every generated state. Therefore, DPSP is applicable to ensemble models by evaluating whether the sampled distribution and its presumed context are sufficient for the designated endpoint.

### 6.4. DPSP Is Not a Sequence-to-Function Predictor

DPSP does not compete with protein language models, annotation systems, generative design algorithms, or structure predictors. These systems make forward predictions from sequence. DPSP begins after a function has been proposed or an endpoint selected and assesses the evidence for that claim. A function annotation produced by a model, or a designed sequence, is not validated merely because a plausible structure is predicted. The sequence may fail to express, fold, remain soluble, assemble, acquire required modifications, or perform the proposed activity under biological conditions. A predicted structure can increase plausibility and guide experiments; it does not convert a probabilistic annotation into an established function.

## 7. Implications for Drug Discovery and Protein Therapeutics

### 7.1. What Structure Contributes Within an Iterative Program

Two assertions must be distinguished. The first asserts that structure solely demonstrates mechanism. This assertion is seldom justifiable and is not posited herein. The second asserts that structure constrains and expedites mechanistic and medicinal-chemistry hypotheses. This assertion is well substantiated and shows how structural information is used in practice.

Contemporary practice reflects the second assertion more closely than the first. Fragment-based discovery uses the structures of weak binders to guide processes such as growth, merging, and linking, thereby enabling the generation of clinical compounds from initial points too weak to be identified through conventional screening methods [80]. Free-energy perturbation predicts relative potency within congeneric series with useful accuracy, provided the binding mode is correct and the relevant state is sufficiently represented. This exemplifies a pertinent case within this framework, as its predictive accuracy depends on the specific conditions DPSP addresses [81]. Cryptic-site detection uses simulation, cosolvent mapping, and fragment docking to identify pockets not visible in the unbound structure, broadening structural design to targets previously deemed undruggable in a single conformation [82]. Allosteric programs use state-specific structural data to design modulators, whose effects are characterized by the state they stabilize. Structure-activity analyses conducted through successive iterative rounds convert each structural hypothesis into measurable outcomes, which then inform and refine subsequent design stages.

In each of these workflows, structure is neither the claim nor its proof. It functions as the hypothesis generator within a cycle that concludes with measurement. This precisely exemplifies what the conditional mode describes, and it should not be misconstrued as criticism. The use of conditionals represents the standard and effective operational mode in structure-enabled drug discovery. The framework is designed to render the necessary corroboration explicit and specific, rather than to discourage structural inquiry.

### 7.2. Geometry-Dominated Endpoints

Structure-based design is most direct when the intervention is defined by a represented pocket or interface, and the design question is geometric. Structures support docking, fragment placement, steric optimization, residue selection, and hypotheses regarding selectivity [83,84]. HIV protease inhibitors serve as an instructive example of structure-assisted design around a well-characterized active site [70]. This success was attributed to the availability of the relevant state and a geometry-oriented design question, combined with iterative biochemical testing, rather than any inherent predictability of the target class.

Even here, the structure functions more as an anchor than as definitive evidence. Docking scores do not establish binding free energy, and a favorable pose can be compromised by desolvation, conformational penalties, competing states, or rapid dissociation. Consequently, evaluate the model against the experimental endpoint used in program optimization.

### 7.3. State-, Kinetic-, and Chemistry-Dependent Endpoints

Allosteric modulators, transporters, ion channels, and numerous kinases operate through mechanisms dependent on conformational states, wherein ligand efficacy is regulated by the populations and interconversions among various conformations. A static complex identifies contact points but need not ascertain whether a ligand stabilizes an active, inactive, desensitized, or biased signaling state. Therefore, direct measurement of activity, state populations, and kinetics is crucial when these factors underpin the therapeutic assertion. Protein–protein interfaces are characterized by interface geometry and quaternary structure [85], whereas binding affinity and residence time at these interfaces are also influenced by conformational and kinetic factors not determined solely by interface geometry.

Binding affinity constitutes a thermodynamic property inherent to the association process rather than an observable characteristic of a single conformation. Surface plasmon resonance differentiates between association and dissociation kinetics, whereas isothermal titration calorimetry and equilibrium techniques offer complementary thermodynamic assessments [86,87]. The influence of flexibility on affinity is context-dependent and cannot be deduced solely from a generic energy difference between two conformations without incorporating a population-weighted binding model [88]. In catalysis, kinetic assays remain essential, as turnover involves chemistry, gating mechanisms, substrate and product exchange, and, in some cases, alternative conformations [65,67].

### 7.4. Product-Specific Physicochemical Properties

In protein therapeutics, the focus is typically on the manufactured product rather than an idealized polypeptide chain. Factors such as glycosylation, oxidation, deamidation, clipping, oligomerization, formulation, concentration, and interfaces significantly influence pharmacokinetics, potency, immunogenicity, and stability. These attributes are closely monitored during developability assessment because they are not determined solely by the amino acid sequence [71]. Glycan composition is particularly critical, since products with similar backbones can differ in clearance rates, receptor interactions, and immune recognition [72]. A deglycosylated or apoprotein model does not adequately represent properties that depend on the actual glycoform or formulation.

Metals and cofactors present an exceptional case, as including the appropriate cofactor improves interpretability of a functional site by delineating coordination geometry and catalytic organization [73]. Membrane composition similarly influences receptor and transporter states. Coarse-grained and multiscale simulations serve as valuable adjuncts for such assemblies, given their ability to extend accessible length and time scales by orders of magnitude and facilitate sampling of large complexes, membrane environments, and slow collective motions beyond the capabilities of all-atom molecular dynamics, as demonstrated by the Martini force field and structure-prediction models such as AWSEM-Suite [89,90]. However, their limitations are equally significant. Reduced representations diminish chemical resolution, outcomes depend on selected parameters and mappings, kinetic data derived from coarse-grained dynamics require careful interpretation, and reverting to atomic detail necessitates back mapping. Consequently, these methods complement all-atom molecular dynamics, enhanced sampling techniques, nuclear magnetic resonance, hydrogen-deuterium exchange, and cryo-electron microscopy. Their results warrant validation against higher-resolution computational or experimental data before they can be considered definitive.

### 7.5. Worked Drug-Development Case: A Kinase Inhibitor Program

A kinase program illustrates how a single target transitions between different modes as the endpoint varies. To design an ATP-competitive inhibitor targeting a well-characterized active-site geometry, a high-quality structure of the relevant kinase conformation is generally adequate, as the program is primarily geometry-driven: the pocket, hinge interactions, and gatekeeper residue are represented, and the model addresses the geometric design issue. When the endpoint shifts to conformational selectivity, such as determining whether a candidate preferentially stabilizes an inactive DFG-out state, reliance on a solitary active-state structure becomes conditional. This is because the decision now depends on the relative populations of active and inactive states and the transition between them, factors that the model does not account for. When the endpoint advances further to cellular potency, residence time, or resistance liability, the program adopts a function-measured approach: a measured dissociation rate, a cellular activity assay, or a mutational panel becomes decisive, and the structure serves to rationalize and guide rather than definitively establish the result. The adequacy of the same coordinate model, whether sufficient, conditional, or inadequate, depends entirely on the specific endpoint and the implications of potential errors for that decision.

## 8. Falsifiability and Validation

Two prospective studies are delineated herein. They are individual investigations with distinct primary endpoints, measurement units, data sources, and refutation criteria, and they are mutually independent. Study 1 evaluates whether DPSP ratings provide information regarding prediction errors associated with mutation-induced alterations in binding free energy. Study 2 assesses whether a singular represented state is sufficient for addressing a bounded pose inquiry. Maintaining their separation is crucial, as a threshold expressed in kilocalories per mole has no meaning for a geometric endpoint, and aggregating the results could lead to a situation where a failure in one study is compensated by a success in the other.

### 8.1. Study 1: Propositions for Mutation-Induced Changes in Binding Free Energy

Study 1 is limited to a single metric, namely the change in protein–protein binding free energy upon mutation, expressed in kilocalories per mole. Binding kinetics are entirely excluded. SKEMPI provides association and dissociation rate constants for a subset of mutations; however, these rate constants are not measured in energy units, cannot be evaluated against a kilocalorie-per-mole criterion, and lack a predefined prediction model or error benchmark like those available for binding free-energy change. Assessing them would necessitate a validated kinetic predictor, a specified error metric in rate or logarithmic-rate units, and a noise threshold from which a minimal effect of interest could be determined, all of which are currently unavailable. Consequently, we designate kinetic endpoints for a separate future investigation conducted independently, and we base no assertions in this article on kinetic data.

- Step 1 states that records containing at least one material dimension rated as uncertain or missing should exhibit an absolute prediction error at least 0.25 kcal/mol greater than those records where all material dimensions are rated as adequate. This comparison is conducted after adjustment for the prespecified covariate set, as delineated in Section 8.3, with a 95% cluster-bootstrap confidence interval excluding zero. The analysis is restricted to material gaps, because the framework assumes that nonmaterial gaps do not influence the evidence supported, as shown in the second kinase row of Table 6, where ensemble and kinetics remain uncertain; however, the pose endpoint remains unaffected, and the assignment remains predominantly geometry-led. A prediction that considers nonmaterial uncertainty would examine a domain not asserted by the framework. Consequently, we analyze records where the only uncertain or missing dimensions are nonmaterial within the adequate group, and we report the total number of such records.
- Step 2 posits that the incorporation of experimentally validated information related to state, context, kinetics, or chemistry is expected to reduce the held-out-family root-mean-square error by at least 0.20 kcal/mol more in dependency-positive groups relative to dependency-negative groups. These groups are delineated based on published experimental evidence and are not defined through DPSP ratings, as elaborated in Section 8.3. The proposition is deemed invalid if the observed interaction is less than 0.20 kcal/mol or if its 95% cluster-bootstrap confidence interval includes zero.

These propositions do not require each protein to be part of a substantial ensemble, nor do they require every dynamic measurement to improve predictive accuracy. They explicitly accommodate a null region wherein supplementary ensemble information provides no additional predictive benefit. This region is integral, as its absence would render the framework unfalsifiable, with all successes attributed to structural factors and all failures attributed to concealed dynamics.

### 8.2. Basis for the Provisional Thresholds

The cutoff values specified above are strategic decisions rather than precise theoretical quantities, and they remain provisional throughout. Two considerations underpin the 0.25 kcal/mol criterion in Proposition 1. Reported uncertainties for mutation-induced alterations in binding free energy within curated benchmarks typically range by a few tenths of a kilocalorie per mole; consequently, an overall difference significantly below approximately 0.2 kcal/mol cannot be distinguished from measurement and curation noise [20]. Structure-based predictors for these changes generally exhibit root-mean-square errors around 1 to 1.5 kcal/mol on curated protein–protein benchmarks, indicating that a 0.25 kcal/mol effect corresponds to roughly one-fifth of the residual error. This magnitude is sufficiently substantial to influence ranking decisions and sufficiently modest to plausibly represent a contribution from an annotation-level covariate [91,92,93].

The 0.20 kcal/mol criterion in Proposition 2 is regarded as a provisional minimum effect of interest rather than a statistical concession. It is established just above the noise floor described earlier and below the primary effect outlined in Proposition 1, based on the judgment that an interaction of this magnitude would influence the prioritization of corroborating evidence while remaining detectable with a feasible sample size. Lower precision in estimating interaction terms is not, by itself, a sufficient reason to consider a smaller effect as meaningful; rather, it is addressed within the context of sample size calculations and the width of the reported confidence interval, rather than by relaxing the criterion. The reliability target of 0.70 for quadratic-weighted agreement aligns with the conventional threshold for substantial categorical agreement [94].

None of these values are derived from theoretical considerations, nor should they be regarded as standards. Both values remain fixed throughout Study 1 and are not revised at any point, whether before or after unblinding. No separate statistical calibration set is designated to reset these values; instead, the 50 records described in Section 8.3 are used to calibrate assessors, not thresholds. If Study 1 indicates that the achievable separation is less than initially assumed, this outcome is documented as is and utilized to determine the minimum effect size of interest for a subsequent validation study, which would then necessitate its own preregistration and sample size calculation. Consequently, recalibration is informed by Study 1 rather than conducted by it, and no criterion within this study is modified once the preregistration has been filed.

### 8.3. Study 1 Design, Covariates, Strata, and Models

The study samples four hundred mutation records from at least forty nonredundant SKEMPI 2.0 complexes, excluding entire protein families from model training [20]. Four assessors with a doctorate in structural biology or protein biophysics, each with at least three years of postdoctoral or equivalent experience, calibrate on fifty records and then rate each dimension as adequate, uncertain, or missing, using Table 4. For each dimension rated as uncertain or missing, assessors additionally record a materiality judgment against the endpoint, applying the materiality rule outlined in Section 5.1, along with the rationale, thereby enabling Proposition 1 to be tested concerning material gaps as specified. Assessors evaluate materiality based on the endpoint definition and the plausible range of the unrepresented variable, rather than on the outcome, which assessors do not observe. Assessors are blinded to measured values, baseline predictions, and outcome labels. The study’s overall reliability target is a kappa coefficient of at least 0.70 for both components: the quadratic-weighted kappa for the three-level dimension ratings, and the unweighted kappa for the derived binary classification of material gaps, which indicates whether the record contains at least one material gap.

One covariate set is prespecified and utilized in all analyses and comparisons documented in this publication: coordinate accuracy of the represented complex, protein structural class, sequence conservation at the mutated position, disorder content of the chain, and reported model confidence where a predicted model is employed. Proposition 1 accounts for this set; the incremental-value analysis examines whether DPSP ratings provide additional information beyond this set, and Table 7 refers to the same set. No covariate is added or removed after assessing the outcome.

A curator who does not participate in DPSP scoring determines dependency status, relying solely on published experimental evidence. DPSP ratings do not delineate either stratum. A record is classified as dependency-positive when the source literature reports experimental evidence indicating that the endpoint depends on a nominated state, context, kinetic factor, or chemical variable. Conversely, a record is deemed dependency-negative when the literature presents experimental evidence suggesting that the endpoint is unaffected by these variables, such as a rigid interface with a specified mutant species structure and documented insensitivity across the relevant conditions. Records for which the literature provides insufficient evidence to support either classification are deemed indeterminate and are subsequently excluded from the Proposition 2 interaction test, with the number of excluded records duly reported.

The baseline is a structure-only predictor of mutation-induced changes in binding free energy applied to the represented complex, using FoldX (version 4) with a documented repair protocol, with Rosetta flex ddG as a predefined secondary baseline [91,93]. The added-evidence model includes the same baseline, supplemented with manually designated evidence variables. Software versions, parameters, the regression specification, the clustering unit for bootstrap, and the management of missing covariates are fixed in the preregistration before any outcome access, with any deviations documented with justifications. The baseline does not incorporate any features derived from DPSP annotation. Both models are calibrated using family-held-out cross-validation, ensuring that a protein family does not appear in both training and testing datasets.

### 8.4. Study 2: A Separate Test of Single-State Adequacy for Bounded Pose Questions

Study 2 constitutes a distinct prospective investigation with a geometric endpoint, and it does not share data, threshold, or analysis model with Study 1. It evaluates Proposition 3: when an independently characterized binding-competent state and the relevant context are represented, a single-state model should be noninferior to the pre-established primary ensemble comparator concerning the bounded pose endpoint. The primary endpoint, binding-pose root-mean-square deviation in angstroms (lower values indicate better performance), is assessed against a single noninferiority margin. The primary comparator employed is the 100 ns explicit-solvent molecular dynamics ensemble, selected a priori as the principal physics-based ensemble comparator. The subsampled-alignment and generative-emulation ensembles are designated as prespecified sensitivity analyses, reported with point estimates and confidence intervals; neither forms the basis for decision-making. Pocket-contact recovery serves as a prespecified secondary endpoint, presented with its point estimate and confidence interval, and is utilized to verify that the primary outcome is not an artifact of pose scoring; no decision rule depends on it, and no multiplicity adjustment is necessary. This proposition remains exploratory until the margin is estimated from a calibration set and fixed prior to unblinding the test set. Once established, it is refuted if the upper bound of the two-sided 95% confidence interval for the mean difference in pose deviation, single-state minus primary-comparator ensemble, exceeds the margin.

The study analyzes the PDBbind 2020 refined set, selecting up to 200 test-ligand complexes with a crystallographic resolution of 2.5 Å or better. These complexes must include a noncovalent ligand comprising 8 to 60 heavy atoms and a binding-competent structure of the same protein, independently characterized and exhibiting at least 95% sequence identity within the same biologically relevant oligomer. Furthermore, the structure should be bound to a different ligand within the same pocket. Protein families do not overlap between calibration and test sets. The methodology employed is cross-ligand docking: the test ligand and its holo coordinates are withheld during docking against the independently characterized structure, as well as against three predetermined 20-conformer ensembles generated from that structure via subsampled-alignment prediction, generative ensemble emulation, and 100 ns explicit-solvent molecular dynamics simulations. We use the holo coordinates solely to calculate the pose root-mean-square deviation and the contact-recovery measure, while fixing the docking software, scoring functions, and versions prior to registration. Eligibility criteria are established before analysis. If fewer than 200 complexes meet the criteria, we use all eligible complexes and classify the study as a feasibility and precision-estimation investigation rather than a powered noninferiority test. If 200 or more complexes qualify, the margin and final sample size are determined from a 50-case calibration set and fixed before unblinding, aiming for 90% statistical power at a one-sided alpha level of 0.025. This is equivalent to the upper bound of a two-sided 95% confidence interval.

Neither study encompasses binding kinetics, catalysis, or signaling; furthermore, a rubric validated solely on binding free-energy change cannot be presumed applicable to these areas. Extending the approach necessitates the independent assembly of specific resources: for kinetics, this includes a validated predictor of association and dissociation rate constants, an error metric expressed in rate or log-rate units, and a noise floor from which a minimum effect of interest can be established; for catalysis, curated datasets of steady-state and pre-steady-state kinetics with matched structures, mutation records, and reaction conditions are required; for signaling, datasets involving dose–response and pathway bias, with explicitly defined receptor constructs, lipid environments, and transducer couplings, are essential. Each extension demands its own annotation manual, dependency definitions, and thresholds, rendering each a distinct area of investigation. One established methodology for constraining transient, low-population states, where a state-resolved reference is necessary, is paramagnetic relaxation enhancement [95].

### 8.5. Preventing Circularity and Outcome Leakage

A validation of an annotation framework may fail in a particular manner: the annotation might encode the outcome it is intended to predict. The design maintains separation between the annotation procedure, the outcome definition, and the predictive features through four safeguards.

- First, a preregistered annotation manual is versioned and published before any scoring begins. It fixes the rubric in Table 4, the evidence admissible for each rating under the evidence rule in Section 5.1, the materiality criteria, the adjudication procedure, the covariate set, the dependency definitions, and the analysis models. Assessors may not modify it during the study, and any necessary amendment is versioned, dated, and reported with the records scored under each version.
- Second, assessors review only the structural representation, the mutation, the endpoint definition, and the source literature evidence pertinent to state, context, kinetics, and chemistry. An independent curator, who does not participate in the scoring process, excludes measured values, baseline predictions, and any textual descriptions of experimental results. Scoring packages are assembled before outcome extraction, and an impartial third party audits a subset of these packages for remaining outcome information, with the audit findings duly reported.
- Third, the dependency status is designated as outlined in Section 8.3, based solely on published experimental evidence pertaining to the variable, and not on the magnitude or direction of the outcome. This status is determined prior to the disclosure of the outcome. Records for which the sole evidence of dependency is the magnitude of the prediction error are classified as indeterminate and are excluded, as their inclusion would be inherently circular in reasoning.
- Fourth, we test the primary result against a preregistered permutation null hypothesis. DPSP ratings are permuted within each protein family, thereby maintaining the marginal distribution of ratings and the family structure whilst eliminating any genuine association. The analysis is subsequently rerun to derive a null distribution of the estimated effect. The observed effect must surpass the 95th percentile of this distribution. A secondary control involves examining the dependency-negative stratum, where the endpoint is documented as insensitive to the nominated variables. Consequently, DPSP ratings in this context should carry no information about prediction error; a positive association in this setting indicates leakage or confounding rather than supporting evidence, prompting re-examination of the data packets and curation process.

### 8.6. Reliability Pilot

A preliminary reliability pilot should precede Study 1, and its design is fixed herein. Three qualified assessors score twenty SKEMPI records spanning at least eight families independently after calibration on ten separate records, using the published manual and blinded packets. The primary outcome is the quadratic-weighted kappa for each dimension, with 95% bootstrap confidence intervals, along with the frequency of one-level and two-level disagreements and the reasons documented during adjudication. Since Proposition 1 classifies records into those containing at least one substantial gap and those with none, agreement regarding materiality is estimated at the record level rather than the dimension level. Each assessor’s per-dimension judgments are condensed into a single binary value per record, indicating the presence or absence of a material gap, ensuring each assessor provides a value for every record regardless of the ratings assigned. Agreement is then calculated as unweighted kappa, with 95% bootstrap confidence intervals derived from individual judgments recorded prior to any consensus or adjudication process. The criterion for proceeding is a lower confidence bound exceeding 0.50 on at least three of the four dimensions and exceeding 0.50 for the record-level material-gap classification; failure to meet this criterion signifies that the rubric requires revision before any predictive claims can be validated. Notably, no pilot has been conducted, and this article reports no pilot results. The contribution presented herein involves reporting a rubric along with detailed conditions under which it would be deemed unreliable; establishing its reliability necessitates the study described above.

### 8.7. What Would Refute the Framework

DPSP would be compromised if accurate coordinates of the relevant state reliably delineated a range of functional endpoints without supplementary information regarding context, populations, kinetics, chemistry, or direct activity. Additionally, it would be compromised if independent assessors could not apply its dimensions consistently, if those dimensions provided no predictive value beyond the predefined covariate set outlined in Section 8.3, or if the results failed to replicate across protein families. Such outcomes should be documented as evidence opposing the framework rather than ascribing them to unobserved dynamics.

Isolated instances where dynamics are demonstrated to be significant do not substantiate the framework. Validation necessitates preregistered endpoints, controls, calibration procedures, and external replication. Until these studies are conducted, the DPSP should be considered an unverified qualitative decision framework, with components derived from established protein science.

## 9. Limitations

DPSP constitutes a qualitative and unvalidated interpretive framework. It does not supply calibrated probabilities nor establish universal thresholds, and it cannot confirm the correctness of a specific functional prediction. Evaluation is particularly challenging when the relevant states or conditions are unknown, and the four dimensions are interconnected rather than independent, which is why they are never combined into a composite score. The rubric in Table 4 remains provisional and is expected to evolve once inter-rater data become available. A single unblinded assessor conducted the illustrative scoring shown in Table 6 on selected cases, and it does not constitute evidence of reliability, predictive validity, or prevalence. The prospective work outlined in Section 8 is confined to two binding endpoints: mutation-induced changes in binding free energy and the bounded pose question, each examined as an individual study. Binding kinetics are excluded from both endpoints due to the absence of validated kinetic predictors, error metrics, or noise floors from which a minimum effect of interest could be derived. No assertion is made that the rubric remains applicable to kinetics, catalysis, signaling, stability, or cellular endpoints.

Placing this framework alongside the Anfinsen and Levinthal paradoxes is expository and does not imply equivalent mechanistic scope, mathematical development, or empirical standing. Whether DPSP earns the term paradox depends on whether its formulation improves inference, experimental design, and prospective prediction beyond existing vocabulary. Earlier work has addressed limitations of static structure prediction, chemical incompleteness, and risk-tiered validation [96,97,98,99]. What is new here is the endpoint-conditioned separation of state accuracy from functional sufficiency, together with a stated rubric and refutation conditions. The authors claim no new prediction algorithm, physical mechanism, or regulatory standard.

The framework should not be employed to rank catalysis, allostery, binding, physicochemical behavior, or therapeutic outcome on a singular universal scale, as these represent different types of phenomena that frequently coexist within a single protein. It should not be utilized to assert that structure-based design is inferior to phenotypic discovery, that protein dynamics account for clinical attrition, or that manipulation of the cellular environment constitutes a validated therapeutic strategy. Such assertions necessitate separate comparative or clinical evidence.

Finally, the term “paradox” is employed in a constrained sense: a practical contradiction that dissipates once the distinction between state accuracy and functional sufficiency is acknowledged. The label is retained because it denotes a recurring practical anomaly: improved coordinates may not necessarily reduce uncertainty about a functional endpoint. The scientific content remains unaffected by the label and can be equivalently expressed as a structure–function underdetermination principle.

## 10. Conclusions

The main inquiry of this article concerns why an accurate depiction of a molecular state may remain insufficient for a condition-specific functional assertion. The resolution involves differentiating structural accuracy from functional adequacy and explicitly articulating the endpoint, the pertinent molecular state, the context, and the critical unconsidered variables prior to deriving a functional conclusion. This delineates an inferential boundary that differs from those established by the Anfinsen and Levinthal paradigms, which elucidate how sequence influences structural predilection and the avoidance of exhaustive search in folding. While the paradigms describe physical phenomena, the boundary discussed herein pertains to what a body of evidence substantively supports; therefore, they should not be regarded as members of a singular category.

DPSP is an evidentiary assessment framework, not an entirely new physical theory or mechanistic model. The framework’s primary function is to organize evidence around a designated functional endpoint. Its contribution is inherently integrative: it consolidates findings long established in structural genomics, function annotation, allostery, disorder biology, and structure-guided discovery into a single endpoint-conditioned procedure. Additionally, it provides a rubric that renders this procedure reproducible in principle and delineates the conditions under which it could be refuted. DPSP complements existing structural, ensemble, and computational methodologies rather than replacing them. Its utility will depend on consistent application, prospective validation, demonstrated value beyond simpler predictors, and confirmation through external replication.

## Figures and Tables

**Table 1:** Relation of DPSP to existing assessment and benchmarking frameworks. This table states where DPSP would be redundant and where it is not.

Existing Framework	What It Evaluates	What It Leaves Unaddressed Here	What DPSP Adds
ASME V&V 40 [17]	Credibility of a computational model against a defined context of use and model risk.	Whether a molecular representation contains the state, partners, populations, and chemistry on which a biological endpoint depends.	A protein-specific decomposition of the evidence gap into relevant state, context, ensemble or kinetics, and chemistry.
FDA framework for AI in regulatory decisions [16]	Fitness of an AI-derived output for a stated regulatory question, scaled to consequence.	What counts as sufficient molecular evidence for a specific structure-to-function claim.	An assessment procedure at the level of the individual claim, with a stated deliverable and three modes of use.
CAFA [18,19]	Accuracy of forward function prediction from sequence and related inputs, scored against ontology terms.	Whether a particular coordinate model supports a particular quantitative endpoint for a particular species and condition.	Evaluation after a claim is made, conditioned on a measurable endpoint rather than an ontology term.
SKEMPI 2.0 and related benchmarks [20]	Predictive accuracy for mutation-induced changes in binding energy, kinetics, and thermodynamics.	Why a given record is predicted poorly, and which missing evidence would improve it.	A prespecified annotation of missing evidence, usable as a covariate and testable against prediction error (Section 8).
Model confidence metrics such as pLDDT and PAE [3,21]	Internal reliability of a coordinate prediction and interface placement.	Population, kinetics, chemistry, and context, none of which these metrics estimate.	An explicit statement that confidence in a representation is not sufficient for a function, with the four dimensions supplying the missing axes.
Integrative ensemble determination and generative ensembles [22,23]	Construction of a distribution of states consistent with data or learned statistics.	Whether the sampled distribution and its assumed context match the endpoint of interest.	Application of the same four dimensions to an ensemble, so that a distribution is assessed rather than assumed adequate.
Structural genomics function-assignment pipelines [11,13]	Transfer of annotation to newly determined structures using templates, homology, and site matching.	Quantitative, condition-specific endpoints such as rate, affinity, residence time, or cellular potency.	A rule for deciding when annotation transfer is adequate evidence and when direct measurement is required.

Abbreviations: DPSP, Dynamic Protein Structure Paradox.

**Table 2:** What a relevant structure can support for common classes of functional question, what remains unresolved, and the corroboration appropriate to the endpoint. This table is indexed by the type of question asked.

Functional Question	What a Relevant Structure Can Support	What Remains Unresolved	Examples of Corroborating Evidence
Fold or domain architecture	Topology, secondary-structure organization, domain boundaries, and steric relationships.	Whether the represented state is populated under the condition of interest, and whether architecture alone implies activity.	Sequence and evolutionary evidence; construct validation; an experimental structure or orthogonal biophysical data where relevant.
Pocket or interface geometry	Shape, residue contacts, electrostatics, and plausible binding modes.	Affinity, kinetics, competition, induced changes, and cellular selectivity.	Binding, competition, mutagenesis, kinetics, or activity assays.
Catalytic mechanism	Catalytic residues, cofactors, substrate orientation, and plausible reaction geometry.	Rate-limiting step, transition-state stabilization, turnover, and specificity across substrates.	Steady-state and pre-steady-state kinetics; isotope effects or other mechanism-specific tests where appropriate.
Allosteric regulation	Candidate effector sites, networks, and state-specific contacts.	State populations, coupling free energies, transition rates, and signaling bias.	Population-sensitive measurements and functional dose–response assays under defined context.
Stability or aggregation	Buried surface, packing defects, exposed hydrophobic regions, and candidate liabilities.	Rates, pathways, colloidal behavior, stress sensitivity, and concentration dependence.	Thermal and chemical stability, aggregation, solubility, and stress studies on the actual construct or product.
Cellular function	A mechanistic hypothesis and residues or interfaces for perturbation.	Localization, expression, partners, regulation, pathway compensation, and phenotype.	Cellular or in vivo assays with target engagement and relevant controls.

**Table 3:** Endpoint definition and the four assessment dimensions used by DPSP. The endpoint definition is a prerequisite; the four rows that follow define sources of potentially missing evidence. This table is indexed by the source of missing evidence rather than by the type of question.

Assessment Element	Question	Evidence to Examine	Interpretive Consequence
Endpoint definition (prerequisite)	Exactly what quantity or behavior is being predicted?	Operational definition, assay readout, molecular species, conditions, decision, and error tolerance.	Prevents a broad structural claim from substituting for a measurable functional claim.
Relevant-state completeness	Does the model contain the state that performs the endpoint?	Conformation, oligomer, ligand, cofactor, metal, mutation, protonation, and post-translational modifications.	A missing state narrows the permitted claim and usually requires additional state-specific evidence.
Context completeness	Are the molecular and environmental selectors of the state represented?	Partners, membrane, pH, ionic and redox conditions, concentration, crowding, and processing.	Missing context makes the prediction conditional on assumptions that must be tested.
Ensemble and kinetic dependence	Do populations or transitions materially control the endpoint?	Exchange and relaxation measurements, hydrogen-deuterium exchange, single-molecule data, cryo-electron microscopy heterogeneity, kinetics, or validated simulations.	High dependence shifts weight from one coordinate model toward population- and rate-sensitive evidence.
Chemical dependence	Does the endpoint require chemistry not encoded by ground-state geometry alone?	Catalytic kinetics, protonation, isotope effects, electrostatics, and solvent and transition-state models.	A ground-state structure can define hypotheses but cannot by itself establish chemical rate or mechanism.

Abbreviations: DPSP, Dynamic Protein Structure Paradox.

**Table 4:** Scoring rubric for the four DPSP dimensions. Ratings are made against a single defined endpoint. Criteria are provisional and are intended to be revised against calibration data.

Dimension	Adequate	Uncertain	Missing
Relevant state	The model contains the conformational and oligomeric state, ligand, cofactor, metal, modification, and protonation assignment required by the endpoint, or an experimental structure of that species exists.	The required species is partly represented, or its identity is inferred from a homolog or from general expectation rather than established for this system.	The model represents a different state or composition from the one the endpoint requires, such as an apo model for a holo function or a monomer for an obligate oligomer.
Context	The conditions that select or stabilize the required state are represented or independently established at the values the endpoint specifies.	One or more selecting conditions are plausible but unverified, or the reported conditions differ from those of the endpoint without evidence that the difference is immaterial.	A condition known to control which state is populated is absent and unspecified, such as an unstated glycoform, lipid environment, redox state, or partner concentration.
Ensemble and kinetics	Population distribution or exchange behavior relevant to the endpoint has been measured, or the endpoint is demonstrably insensitive to redistribution within the accessible ensemble.	Heterogeneity is suspected on structural or sequence grounds, or a simulation suggests it, but no independent observable constrains the populations or rates.	The endpoint depends on a low-population state, on a transition rate, or on residence time, and no population- or rate-resolved evidence exists.
Chemistry	The chemical step is independently characterized, or the endpoint does not depend on proton transfer, electrostatic preorganization, solvent reorganization, or transition-state stabilization.	A chemical mechanism is proposed from geometry alone and is consistent with the structure but has not been tested by kinetic or mechanism-specific measurement.	The endpoint is a chemical rate or mechanism-dependent quantity and only ground-state geometry is available.

Abbreviations: DPSP, Dynamic Protein Structure Paradox.

**Table 5:** Positive, negative, and ambiguous examples for each dimension, with the rule that resolves the ambiguous case. Examples describe representations and endpoints and are not assessments of specific published claims.

Dimension	Positive Example (Adequate)	Negative Example (Missing)	Ambiguous Example and Its Resolution
Relevant state	Endpoint is the binding pose of a congeneric ligand series in a pocket for which a co-crystal structure of the same protein with a close analog exists.	Endpoint is effector function of a therapeutic antibody, and the available model is a deglycosylated backbone.	A high-confidence predicted model of an obligate dimer is provided as a monomer, with the interface predicted separately. Resolution: rate the dimension as uncertain rather than missing if the interface prediction is supported by an experimental structure of a close homolog, and as missing if the oligomeric state itself is in question, since the assembled species is the one that performs the endpoint.
Context	Endpoint is a dissociation constant measured in a defined buffer, and the structure was determined under matched pH and ionic conditions for the same construct.	Endpoint is transporter conformational preference and the model carries no lipid, no membrane potential, and no stated lipid composition.	A kinase structure was determined at pH 6.5 while the cellular endpoint applies near pH 7.2. Resolution: rate the dimension as uncertain, then apply the materiality rule, since the difference is immaterial unless a titratable group in the functional site changes protonation across that range.
Ensemble and kinetics	Endpoint is a bounded pose question in a rigid interface for which room-temperature crystallography shows no alternative backbone conformations in the contact region.	Endpoint is residence time and the only evidence is a single static complex.	A loop adjacent to the site has low predicted confidence and elevated crystallographic B-factors. Resolution: rate the dimension as uncertain rather than missing, since neither observable constrains populations or rates, and escalate to missing only if the endpoint depends on the loop conformation.
Chemistry	Endpoint is steric complementarity of a scaffold in a pocket whose catalytic chemistry is not involved in the claim.	Endpoint is turnover number and only a ground-state model with modeled substrate is available.	A metal site is modeled with the correct coordination geometry but with unverified metal identity. Resolution: rate chemistry as uncertain and relevant state as uncertain, and target corroboration at the upstream variable by establishing metal identity and occupancy first, per Section 4.5.

**Table 6:** Illustrative application of the rubric to nine endpoint and representation pairs. Author-scored, unblinded, and selected to span the assignment space. Each row is scored against the representation named in that row rather than against all published evidence for the system. This demonstrates rubric mechanics and is not evidence of reliability, predictive value, or prevalence.

Endpoint and Available Representation	State	Context	Ensemble and Kinetics	Chemistry	Mode and Next Decisive Measurement
Suitability of the S1 and S1′ subsite geometry of HIV-1 protease for a peptidomimetic scaffold, using a co-crystal structure with a related inhibitor [70]	Adequate	Adequate	Adequate	Adequate	Geometry-led. No material gap; confirm by synthesis and enzyme inhibition within the design cycle.
Hinge-binding geometry for an ATP-competitive kinase inhibitor, using a high-resolution active-state structure of the same kinase	Adequate	Adequate	Uncertain	Adequate	Geometry-led. Loop flexibility adjacent to the site is not material to the pose question; revisit if selectivity becomes the endpoint.
Preferential stabilization of the inactive DFG-out state of the same kinase by a candidate, using the same active-state structure	Uncertain	Adequate	Missing	Adequate	Conditional. Obtain a state-resolved structure or population-sensitive measurement of the DFG-out fraction with and without ligand.
Competence of KaiB to bind KaiC, using a ground-state fold model [68,69]	Missing	Uncertain	Missing	Adequate	Conditional. Obtain state-resolved evidence for the fold-switched conformation, or a partner-bound structure.
Whether a candidate is an inverse agonist that suppresses basal signaling of a G-protein-coupled receptor, using an active-state complex only [53]	Missing	Uncertain	Missing	Adequate	Function-measured. The endpoint is a signaling output, so a dose–response assay under defined lipid and partner conditions is decisive; state-resolved structures would inform but not establish it.
Whether the same candidate preferentially occupies the inactive-state ensemble of that receptor, using the same active-state complex [53]	Missing	Uncertain	Missing	Adequate	Conditional. The endpoint is a state population, which an inactive-state structure or a population-resolved measurement can presently supply.
Fc-receptor-dependent effector function of a therapeutic IgG1, using a deglycosylated backbone model [71,72]	Missing	Missing	Uncertain	Adequate	Function-measured. Determine the glycoform distribution of the actual product and measure receptor binding and cell-based effector activity.
Support for a proposed two-metal mechanism in a metalloenzyme, using a ground-state structure with unverified metal identity [63,73]	Uncertain	Uncertain	Adequate	Missing	Function-measured. Establish metal identity and occupancy first, then measure kinetics and mechanism-specific observables.
Whether a motif-containing disordered region contributes to the interface used for binding to a named partner, given that binding of the full-length proteins is already established, using a model in which the region is unassigned [44,45]	Missing	Uncertain	Missing	Adequate	Conditional. The endpoint is which segment forms the interface, which a complex structure of the bound motif or a peptide-competition experiment can presently establish.

Ratings follow Table 4. Mode assignment follows the ordered rule in Section 4.6.

**Table 7:** Testable DPSP propositions, the respective studies to which they belong, prospective tests, primary analyses, and outcomes that could potentially weaken or refute them. Propositions 1 and 2 are associated with Study 1 and are expressed in kilocalories per mole; Proposition 3 is associated with Study 2 and adopts a geometric form. Kinetic endpoints are not evaluated in either study.

Proposition and Study	Prospective Test	Primary Analysis	Result That Would Weaken or Refute the Proposition
Proposition 1 (Study 1). A material dimension rated uncertain or missing increases absolute prediction error for mutation-induced change in binding free energy by at least 0.25 kcal/mol.	In 400 SKEMPI 2.0 records from at least 40 family-held-out complexes, compare baseline errors between records with at least one material gap and records whose material dimensions are all adequate, with nonmaterial gaps analyzed in the latter group.	Difference adjusted for the prespecified covariate set in Section 8.3; criterion at least 0.25 kcal/mol with a 95% cluster-bootstrap confidence interval excluding zero, and an effect exceeding the 95th percentile of the within-family permutation null.	Difference below 0.25 kcal/mol, confidence interval including zero, effect within the permutation null, or no improvement over the covariate set alone.
Proposition 2 (Study 1). Added evidence improves prediction more for dependency-positive than for dependency-negative endpoints.	Before outcome disclosure, an independent curator assigns dependency status from published experimental evidence; compare added-evidence and baseline models across strata, excluding indeterminate records.	Interaction in held-out-family error improvement; criterion at least 0.20 kcal/mol with a 95% cluster-bootstrap confidence interval excluding zero.	Interaction below 0.20 kcal/mol, confidence interval including zero, equal improvement in both strata, or association detected in the dependency-negative stratum, which indicates leakage.
Proposition 3 (Study 2). Single-state models are adequate for bounded geometry-dominated questions when an independently characterized binding-competent state is represented.	Cross-ligand docking of up to 200 eligible PDBbind 2020 refined-set complexes: one binding-competent structure with a different pocket ligand versus prespecified ensembles, with test-ligand holo coordinates withheld.	Paired noninferiority on the primary endpoint, binding-pose root-mean-square deviation, against the prespecified primary comparator, the molecular dynamics ensemble, at one-sided alpha 0.025; single margin and sample size fixed after calibration and before test unblinding. Pocket-contact recovery and the other two ensembles are reported as supportive analyses with no decision rule. If fewer than 200 cases qualify, report feasibility and precision only.	After the margin is fixed, the upper bound of the two-sided 95% confidence interval for the mean difference in pose deviation, single-state minus primary-comparator ensemble, exceeds the margin.
Reliability check (precedes Study 1). Not a proposition: DPSP ratings can be applied consistently and add information beyond simpler labels.	Pilot of 20 records with three assessors (Section 8.6), followed by four blinded assessors in Study 1 after calibration on 50 records.	Quadratic-weighted kappa per dimension and unweighted kappa for the record-level material-gap classification, each with 95% bootstrap confidence intervals, computed from individual judgments; target at least 0.70 for both in Study 1; incremental value assessed beyond the covariate set in Section 8.3.	Failure to reach the reliability target on the dimension ratings or on the record-level material-gap classification, failure to replicate externally, or no improvement over the covariate set.

Abbreviations: DPSP, Dynamic Protein Structure Paradox.

## Data Availability

No new data were created or analyzed in this study. Data sharing is not applicable to this article.

## References

[1] Kendrew J.C., Bodo G., Dintzis H.M., Parrish R.G., Wyckoff H., Phillips D.C. (1958). A three-dimensional model of the myoglobin molecule obtained by X-ray analysis. Nature.

[2] Berman H.M., Westbrook J., Feng Z., Gilliland G., Bhat T.N., Weissig H., Shindyalov I.N., Bourne P.E. (2000). The Protein Data Bank. Nucleic Acids Res..

[3] Jumper J., Evans R., Pritzel A., Green T., Figurnov M., Ronneberger O., Tunyasuvunakool K., Bates R., Zidek A., Potapenko A. (2021). Highly accurate protein structure prediction with AlphaFold. Nature.

[4] Baek M., DiMaio F., Anishchenko I., Dauparas J., Ovchinnikov S., Lee G.R., Wang J., Cong Q., Kinch L.N., Schaeffer R.D. (2021). Accurate prediction of protein structures and interactions using a three-track neural network. Science.

[5] Lin Z., Akin H., Rao R., Hie B., Zhu Z., Lu W., Smetanin N., Verkuil R., Kabeli O., Shmueli Y. (2023). Evolutionary-scale prediction of atomic-level protein structure with a language model. Science.

[6] Abramson J., Adler J., Dunger J., Evans R., Green T., Pritzel A., Ronneberger O., Willmore L., Ballard A.J., Bambrick J. (2024). Accurate structure prediction of biomolecular interactions with AlphaFold 3. Nature.

[7] Whisstock J.C., Lesk A.M. (2003). Prediction of protein function from protein sequence and structure. Q. Rev. Biophys..

[8] Orengo C.A., Jones D.T., Thornton J.M. (1994). Protein superfamilies and domain superfolds. Nature.

[9] Todd A.E., Orengo C.A., Thornton J.M. (2001). Evolution of function in protein superfamilies, from a structural perspective. J. Mol. Biol..

[10] Jeffery C.J. (1999). Moonlighting proteins. Trends Biochem. Sci..

[11] Grabowski M., Niedzialkowska E., Zimmerman M.D., Minor W. (2016). The impact of structural genomics: The first quindecennial. J. Struct. Funct. Genom..

[12] Burley S.K., Joachimiak A., Montelione G.T., Wilson I.A. (2008). Contributions to the NIH-NIGMS Protein Structure Initiative from the PSI Production Centers. Structure.

[13] Watson J.D., Laskowski R.A., Thornton J.M. (2005). Predicting protein function from sequence and structural data. Curr. Opin. Struct. Biol..

[14] Anfinsen C.B. (1973). Principles that govern the folding of protein chains. Science.

[15] Levinthal C., Debrunner P., Tsibris J., Munck E. (1969). How to fold graciously. Mossbauer Spectroscopy in Biological Systems.

[16] U.S. Food and Drug Administration (2025). Considerations for the Use of Artificial Intelligence to Support Regulatory Decision-Making for Drug and Biological Products: Draft Guidance for Industry and Other Interested Parties.

[17] (2018). Assessing Credibility of Computational Modeling Through Verification and Validation: Application to Medical Devices.

[18] Radivojac P., Clark W.T., Oron T.R., Schnoes A.M., Wittkop T., Sokolov A., Graim K., Funk C., Verspoor K., Ben-Hur A. (2013). A large-scale evaluation of computational protein function prediction. Nat. Methods.

[19] De Paolis Kaluza M.C., Ramola R., Joshi P., Piovesan D., Reade W., Orchard S., Martin M.J., Ignatchenko A., Rost B., Orengo C.A. (2026). Advances in Protein Function Prediction from the Fifth CAFA Challenge. bioRxiv.

[20] Jankauskaite J., Jimenez-Garcia B., Dapkunas J., Fernandez-Recio J., Moal I.H. (2019). SKEMPI 2.0: An updated benchmark of changes in protein-protein binding energy, kinetics and thermodynamics upon mutation. Bioinformatics.

[21] Akdel M., Pires D.E.V., Pardo E.P., Janes J., Zalevsky A.O., Meszaros B., Bryant P., Good L.L., Laskowski R.A., Pozzati G. (2022). A structural biology community assessment of AlphaFold2 applications. Nat. Struct. Mol. Biol..

[22] Bonomi M., Heller G.T., Camilloni C., Vendruscolo M. (2017). Principles of protein structural ensemble determination. Curr. Opin. Struct. Biol..

[23] Lewis S., Hempel T., Jimenez-Luna J., Gastegger M., Xie Y., Foong A.Y.K., Satorras V.G., Abdin O., Veeling B.S., Zaporozhets I. (2025). Scalable emulation of protein equilibrium ensembles with generative deep learning. Science.

[24] Dill K.A., MacCallum J.L. (2012). The protein-folding problem, 50 years on. Science.

[25] Bryngelson J.D., Onuchic J.N., Socci N.D., Wolynes P.G. (1995). Funnels, pathways, and the energy landscape of protein folding: A synthesis. Proteins.

[26] Onuchic J.N., Wolynes P.G. (2004). Theory of protein folding. Curr. Opin. Struct. Biol..

[27] Frauenfelder H., Sligar S.G., Wolynes P.G. (1991). The energy landscapes and motions of proteins. Science.

[28] Henzler-Wildman K., Kern D. (2007). Dynamic personalities of proteins. Nature.

[29] Niazi S.K. (2025). Quantum Mechanics Paradox in Protein Structure Prediction: Intrinsically Linked to Sequence Yet Independent of It. Comput. Struct. Biotechnol. Rep..

[30] Niazi S.K. (2025). Critical Assessment of AI-Based Protein Structure Prediction: Fundamental Challenges and Future Directions in Drug Discovery. Comput. Struct. Biotechnol. Rep..

[31] Boehr D.D., Nussinov R., Wright P.E. (2009). The role of dynamic conformational ensembles in biomolecular recognition. Nat. Chem. Biol..

[32] Marks D.S., Colwell L.J., Sheridan R., Hopf T.A., Pagnani A., Zecchina R., Sander C. (2011). Protein 3D structure computed from evolutionary sequence variation. PLoS ONE.

[33] Morcos F., Pagnani A., Lunt B., Bertolino A., Marks D.S., Sander C., Zecchina R., Onuchic J.N., Hwa T., Weigt M. (2011). Direct-coupling analysis of residue coevolution captures native contacts across many protein families. Proc. Natl. Acad. Sci. USA.

[34] Ovchinnikov S., Park H., Varghese N., Huang P.S., Pavlopoulos G.A., Kim D.E., Kamisetty H., Kyrpides N.C., Baker D. (2017). Protein structure determination using metagenome sequence data. Science.

[35] Hopf T.A., Ingraham J.B., Poelwijk F.J., Scharfe C.P.I., Springer M., Sander C., Marks D.S. (2017). Mutation effects predicted from sequence co-variation. Nat. Biotechnol..

[36] Mittermaier A., Kay L.E. (2006). New tools provide new insights in NMR studies of protein dynamics. Science.

[37] Konermann L., Pan J., Liu Y.H. (2011). Hydrogen exchange mass spectrometry for studying protein structure and dynamics. Chem. Soc. Rev..

[38] Ferrari A.J.R., Dixit S.M., Thibeault J., Garcia M., Houliston S., Ludwig R.W., Notin P., Phoumyvong C.M., Martell C.M., Jung M.D. (2026). Large-scale discovery, analysis and design of protein energy landscapes. Nature.

[39] Schuler B., Hofmann H. (2013). Single-molecule spectroscopy of protein folding dynamics: Expanding scope and timescales. Curr. Opin. Struct. Biol..

[40] Fraser J.S., van den Bedem H., Samelson A.J., Lang P.T., Holton J.M., Echols N., Alber T. (2011). Accessing protein conformational ensembles using room-temperature X-ray crystallography. Proc. Natl. Acad. Sci. USA.

[41] Punjani A., Fleet D.J. (2021). 3D variability analysis: Resolving continuous flexibility and discrete heterogeneity from single-particle cryo-EM. J. Struct. Biol..

[42] Shaw D.E., Maragakis P., Lindorff-Larsen K., Piana S., Dror R.O., Eastwood M.P., Bank J.A., Jumper J.M., Salmon J.K., Shan Y. (2010). Atomic-level characterization of the structural dynamics of proteins. Science.

[43] Laio A., Parrinello M. (2002). Escaping free-energy minima. Proc. Natl. Acad. Sci. USA.

[44] Van Roey K., Uyar B., Weatheritt R.J., Dinkel H., Seiler M., Budd A., Gibson T.J., Davey N.E. (2014). Short linear motifs: Ubiquitous and functionally diverse protein interaction modules directing cell regulation. Chem. Rev..

[45] Mohan A., Oldfield C.J., Radivojac P., Vacic V., Cortese M.S., Dunker A.K., Uversky V.N. (2006). Analysis of molecular recognition features (MoRFs). J. Mol. Biol..

[46] Wright P.E., Dyson H.J. (2015). Intrinsically disordered proteins in cellular signalling and regulation. Nat. Rev. Mol. Cell Biol..

[47] Martin E.W., Holehouse A.S., Peran I., Farag M., Incicco J.J., Bremer A., Grace C.R., Soranno A., Pappu R.V., Mittag T. (2020). Valence and patterning of aromatic residues determine the phase behavior of prion-like domains. Science.

[48] Banani S.F., Lee H.O., Hyman A.A., Rosen M.K. (2017). Biomolecular condensates: Organizers of cellular biochemistry. Nat. Rev. Mol. Cell Biol..

[49] Necci M., Piovesan D., Tosatto S.C.E., CAID Predictors, DisProt Curators (2021). Critical assessment of protein intrinsic disorder prediction. Nat. Methods.

[50] Ruff K.M., Pappu R.V. (2021). AlphaFold and implications for intrinsically disordered proteins. J. Mol. Biol..

[51] Monod J., Wyman J., Changeux J.P. (1965). On the nature of allosteric transitions: A plausible model. J. Mol. Biol..

[52] Changeux J.P., Edelstein S.J. (2005). Allosteric mechanisms of signal transduction. Science.

[53] Latorraca N.R., Venkatakrishnan A.J., Dror R.O. (2017). GPCR dynamics: Structures in motion. Chem. Rev..

[54] Chakravarty D., Porter L.L. (2022). AlphaFold2 fails to predict protein fold switching. Protein Sci..

[55] Ellis R.J. (2001). Macromolecular crowding: Obvious but underappreciated. Trends Biochem. Sci..

[56] Rivas G., Minton A.P. (2016). Macromolecular crowding in vitro, in vivo, and in between. Trends Biochem. Sci..

[57] Hartl F.U., Bracher A., Hayer-Hartl M. (2011). Molecular chaperones in protein folding and proteostasis. Nature.

[58] Inomata K., Ohno A., Tochio H., Isogai S., Tenno T., Nakase I., Takeuchi T., Futaki S., Ito Y., Hiroaki H. (2009). High-resolution multi-dimensional NMR spectroscopy of proteins in human cells. Nature.

[59] Banci L., Barbieri L., Bertini I., Luchinat E., Secci E., Zhao Y., Aricescu A.R. (2013). Atomic-resolution monitoring of protein maturation in live human cells by NMR. Nat. Chem. Biol..

[60] Tsai Y.X., Chang N.E., Reuter K., Chang H.T., Yang T.J., von Bulow S., Sehrawat V., Zerrouki N., Tuffery M., Gecht M. (2024). Rapid simulation of glycoprotein structures by grafting and steric exclusion of glycan conformer libraries. Cell.

[61] Rohs R., Jin X., West S.M., Joshi R., Honig B., Mann R.S. (2010). Origins of specificity in protein-DNA recognition. Annu. Rev. Biochem..

[62] Phillips R., Ursell T., Wiggins P., Sens P. (2009). Emerging roles for lipids in shaping membrane-protein function. Nature.

[63] Rosato A., Valasatava Y., Andreini C. (2016). Minimal functional sites in metalloproteins and their usage in structural bioinformatics. Int. J. Mol. Sci..

[64] Dobson C.M. (2003). Protein folding and misfolding. Nature.

[65] Hammes-Schiffer S., Benkovic S.J. (2006). Relating protein motion to catalysis. Annu. Rev. Biochem..

[66] Warshel A. (1998). Electrostatic origin of the catalytic power of enzymes. J. Biol. Chem..

[67] Fraser J.S., Clarkson M.W., Degnan S.C., Erion R., Kern D., Alber T. (2009). Hidden alternative structures of proline isomerase essential for catalysis. Nature.

[68] Chang Y.G., Cohen S.E., Phong C., Myers W.K., Kim Y.I., Tseng R., Lin J., Zhang L., Boyd J.S., Lee Y. (2015). A protein fold switch joins the circadian oscillator to clock output in cyanobacteria. Science.

[69] Snijder J., Schuller J.M., Wiegard A., Lossl P., Schmelling N., Axmann I.M., Plitzko J.M., Forster F., Heck A.J.R. (2017). Structures of the cyanobacterial circadian oscillator frozen in a fully assembled state. Science.

[70] Wlodawer A., Vondrasek J. (1998). Inhibitors of HIV-1 protease: A major success of structure-assisted drug design. Annu. Rev. Biophys. Biomol. Struct..

[71] Xu Y., Wang D., Mason B., Rossomando T., Li N., Liu D., Cheung J.K., Xu W., Raghava S., Katiyar A. (2019). Structure, heterogeneity and developability assessment of therapeutic antibodies. mAbs.

[72] Zhou Q., Qiu H. (2019). The mechanistic impact of N-glycosylation on stability, pharmacokinetics, and immunogenicity of therapeutic proteins. J. Pharm. Sci..

[73] Andreini C., Bertini I., Cavallaro G., Holliday G.L., Thornton J.M. (2008). Metal ions in biological catalysis: From enzyme databases to general principles. J. Biol. Inorg. Chem..

[74] Varadi M., Anyango S., Deshpande M., Nair S., Natassia C., Yordanova G., Yuan D., Stroe O., Wood G., Laydon A. (2022). AlphaFold Protein Structure Database: Massively expanding the structural coverage of protein-sequence space with high-accuracy models. Nucleic Acids Res..

[75] Tunyasuvunakool K., Adler J., Wu Z., Green T., Zielinski M., Zidek A., Bridgland A., Cowie A., Meyer C., Laydon A. (2021). Highly accurate protein structure prediction for the human proteome. Nature.

[76] Saldano T., Escobedo N., Marchetti J., Zea D.J., Mac Donagh J., Velez Rueda A.J., Gonik E., Garcia Melani A., Novomisky Nechcoff J., Salas M.N. (2022). Impact of protein conformational diversity on AlphaFold predictions. Bioinformatics.

[77] Tejero R., Huang Y.J., Ramelot T.A., Montelione G.T. (2022). AlphaFold models of small proteins rival the accuracy of solution NMR structures. Front. Mol. Biosci..

[78] Fowler N.J., Williamson M.P. (2022). The accuracy of protein structures in solution determined by AlphaFold and NMR. Structure.

[79] del Alamo D., Sala D., Mchaourab H.S., Meiler J. (2022). Sampling alternative conformational states of transporters and receptors with AlphaFold2. eLife.

[80] Erlanson D.A., Fesik S.W., Hubbard R.E., Jahnke W., Jhoti H. (2016). Twenty years on: The impact of fragments on drug discovery. Nat. Rev. Drug Discov..

[81] Wang L., Wu Y., Deng Y., Kim B., Pierce L., Krilov G., Lupyan D., Robinson S., Dahlgren M.K., Greenwood J. (2015). Accurate and reliable prediction of relative ligand binding potency in prospective drug discovery by way of a modern free-energy calculation protocol and force field. J. Am. Chem. Soc..

[82] Vajda S., Beglov D., Wakefield A.E., Egbert M., Whitty A. (2018). Cryptic binding sites on proteins: Definition, detection, and druggability. Curr. Opin. Chem. Biol..

[83] Sliwoski G., Kothiwale S., Meiler J., Lowe E.W. (2014). Computational methods in drug discovery. Pharmacol. Rev..

[84] Kitchen D.B., Decornez H., Furr J.R., Bajorath J. (2004). Docking and scoring in virtual screening for drug discovery. Nat. Rev. Drug Discov..

[85] Janin J., Bahadur R.P., Chakrabarti P. (2008). Protein-protein interaction and quaternary structure. Q. Rev. Biophys..

[86] Rich R.L., Myszka D.G. (2000). Advances in surface plasmon resonance biosensor analysis. Curr. Opin. Biotechnol..

[87] Velazquez-Campoy A., Freire E. (2006). Isothermal titration calorimetry to determine association constants for high-affinity ligands. Nat. Protoc..

[88] Gilson M.K., Zhou H.X. (2007). Calculation of protein-ligand binding affinities. Annu. Rev. Biophys. Biomol. Struct..

[89] Souza P.C.T., Alessandri R., Barnoud J., Thallmair S., Faustino I., Grunewald F., Patmanidis I., Abdizadeh H., Bruininks B.M.H., Wassenaar T.A. (2021). Martini 3: A general purpose force field for coarse-grained molecular dynamics. Nat. Methods.

[90] Jin S., Chen M., Chen X., Bueno C., Lu W., Schafer N.P., Lin X., Onuchic J.N., Wolynes P.G. (2020). Protein structure prediction in CASP13 using AWSEM-Suite. J. Chem. Theory Comput..

[91] Schymkowitz J., Borg J., Stricher F., Nys R., Rousseau F., Serrano L. (2005). The FoldX web server: An online force field. Nucleic Acids Res..

[92] Kellogg E.H., Leaver-Fay A., Baker D. (2011). Role of conformational sampling in computing mutation-induced changes in protein structure and stability. Proteins.

[93] Barlow K.A., Conchuir S.O., Thompson S., Suresh P., Lucas J.E., Heinonen M., Kortemme T. (2018). Flex ddG: Rosetta ensemble-based estimation of changes in protein-protein binding affinity upon mutation. J. Phys. Chem. B.

[94] Landis J.R., Koch G.G. (1977). The measurement of observer agreement for categorical data. Biometrics.

[95] PDBbind+ PDBbind Database, Version 2020 Refined Set (v.2020R1). https://www.pdbbind-plus.org.cn/download.

[96] Clore G.M., Iwahara J. (2009). Theory, practice, and applications of paramagnetic relaxation enhancement for the characterization of transient low-population states of biological macromolecules and their complexes. Chem. Rev..

[97] Niazi S.K., Mariam Z., Paracha R.Z. (2024). Limitations of Protein Structure Prediction Algorithms in Therapeutic Protein Development. BioMedInformatics.

[98] Niazi S.K. (2026). Quantum Mechanics in Drug Design: Progress, Challenges, and Future Frontiers. Commun. Integr. Biol..

[99] Niazi S.K. (2026). A Risk-Tiered Validation Framework for Artificial Intelligence in Drug Discovery: From Reproducibility to Clinical Translation. Int. J. Mol. Sci..

